# Psychological interventions for common mental disorders in women experiencing intimate partner violence in low-income and middle-income countries: a systematic review and meta-analysis

**DOI:** 10.1016/S2215-0366(19)30510-3

**Published:** 2020-02

**Authors:** Roxanne C Keynejad, Charlotte Hanlon, Louise M Howard

**Affiliations:** a Section of Women's Mental Health, Institute of Psychiatry, Psychology & Neuroscience, King's College London, London, UK; b Centre for Global Mental Health, Institute of Psychiatry, Psychology & Neuroscience, King's College London, London, UK; c Health Service and Population Research Department, Institute of Psychiatry, Psychology & Neuroscience, King's College London, London, UK; d World Health Organization Collaborating Centre for Mental Health Research and Capacity-Building, Department of Psychiatry, School of Medicine, College of Health Sciences, Addis Ababa University, Addis Ababa, Ethiopia; e Centre for Innovative Drug Development and Therapeutic Trials for Africa (CDT-Africa), College of Health Sciences, Addis Ababa University, Addis Ababa, Ethiopia

## Abstract

**Background:**

Evidence on the effectiveness of psychological interventions for women with common mental disorders (CMDs) who also experience intimate partner violence is scarce. We aimed to test our hypothesis that exposure to intimate partner violence would reduce intervention effectiveness for CMDs in low-income and middle-income countries (LMICs).

**Methods:**

For this systematic review and meta-analysis, we searched MEDLINE, Embase, PsycINFO, Web of Knowledge, Scopus, CINAHL, LILACS, ScieELO, Cochrane, PubMed databases, trials registries, 3ie, Google Scholar, and forward and backward citations for studies published between database inception and Aug 16, 2019. All randomised controlled trials (RCTs) of psychological interventions for CMDs in LMICs which measured intimate partner violence were included, without language or date restrictions. We approached study authors to obtain unpublished aggregate subgroup data for women who did and did not report intimate partner violence. We did separate random-effects meta-analyses for anxiety, depression, post-traumatic stress disorder (PTSD), and psychological distress outcomes. Evidence from randomised controlled trials was synthesised as differences between standardised mean differences (SMDs) for change in symptoms, comparing women who did and who did not report intimate partner violence via random-effects meta-analyses. The quality of the evidence was assessed with the Cochrane risk of bias tool. This study is registered on PROSPERO, number CRD42017078611.

**Findings:**

Of 8122 records identified, 21 were eligible and data were available for 15 RCTs, all of which had a low to moderate risk of overall bias. Anxiety (five interventions, 728 participants) showed a greater response to intervention among women reporting intimate partner violence than among those who did not (difference in standardised mean differences [dSMD] 0·31, 95% CI 0·04 to 0·57, *I*^2^=49·4%). No differences in response to intervention were seen in women reporting intimate partner violence for PTSD (eight interventions, n=1436; dSMD 0·14, 95% CI −0·06 to 0·33, *I*^2^=42·6%), depression (12 interventions, n=2940; 0·10, −0·04 to 0·25, *I*^2^=49·3%), and psychological distress (four interventions, n=1591; 0·07, −0·05 to 0·18, *I*^2^=0·0%, p=0·681).

**Interpretation:**

Psychological interventions treat anxiety effectively in women with current or recent intimate partner violence exposure in LMICs when delivered by appropriately trained and supervised health-care staff, even when not tailored for this population or targeting intimate partner violence directly. Future research should investigate whether adapting evidence-based psychological interventions for CMDs to address intimate partner violence enhances their acceptability, feasibility, and effectiveness in LMICs.

**Funding:**

UK National Institute for Health Research ASSET and King's IoPPN Clinician Investigator Scholarship.

## Introduction

The fifth UN sustainable development goal, which is to achieve gender equality and empower all women and girls, emphasises the need to address intimate partner violence.^1^ Intimate partner violence is behaviour by a partner or ex-partner that causes physical, sexual, or psychological harm and includes physical aggression, sexual coercion, psychological abuse, and controlling activity.^2^ It is highly prevalent in low-income and middle-income countries (LMICs); a multi-country study of more than 24 000 women found that lifetime prevalence of physical or sexual intimate partner violence ranged from 24% in urban Serbia and Montenegro to 71% in rural Ethiopia.^3^ Although the availability of national statistics from high-income countries (HICs) is variable, lifetime prevalence of intimate partner violence in LMICs appears to be higher than countries such as Australia (17%) and the UK (29%).^4^

Intimate partner violence is an important social determinant of health.^5^ The association between intimate partner violence and mental health is bidirectional, such that intimate partner violence increases the risk of mental health conditions, which themselves increase vulnerability to intimate partner violence. Intimate partner violence is associated with development of anxiety, depression, and suicide attempts, which can predict subsequent intimate partner violence.^6^ However, there have been concerns that the medical model adopted by mental health services might be counterproductive. Research with survivors of intimate partner violence suggests that failure by mental health services to acknowledge the role and effect of abuse^7^ or to meet women's complex needs^8^ can pathologise intimate partner violence-related difficulties^9^ and reduce the therapeutic potential of treatments.^10^

**Research in context**

**Evidence before this study**

We searched MEDLINE, Embase, PsycINFO, Web of Knowledge, Scopus, CINAHL, LILACS, ScieELO, Cochrane, PubMed databases, trials registries, 3ie, Google Scholar, and forward and backward citations for studies published between database inception and Aug 16, 2019, using search terms pertaining to “randomised controlled trials”, “psychological interventions”, “common mental disorders”, and “low-income and middle-income countries”, without language restrictions. Studies were included if women participated and intimate partner violence exposure was measured. There is scarce evidence on the effectiveness of psychological interventions for women experiencing common mental disorders in the context of intimate partner violence—defined as physical and psychological abuse, sexual coercion, and controlling behaviour by a partner or ex-partner—especially from low and middle-income countries (LMICs). Most published mental health research does not analyse sex and gender differences or the role of gendered risk factors, such as intimate partner violence. There is a growing evidence base for the effectiveness of brief, task-shared psychological interventions for common mental disorders, such as depression and post-traumatic stress disorder (PTSD), in LMICs, but meta-analyses have not reported moderator analyses owing to infrequent measurement and reporting of intimate partner violence.

**Added value of this study**

Despite the well established bidirectional association between intimate partner violence and mental health, this analysis is the first exploration of the moderating effect of intimate partner violence on the effectiveness of treatment for common mental disorders. We found greater improvements in anxiety after psychological intervention among women reporting intimate partner violence exposure than among women who did not report such violence and no differences for depression, PTSD, and psychological distress.

**Implications of all the available evidence**

Women experiencing intimate partner violence face a range of personal, clinician, and health system barriers to accessing mental health care. Our results provide some reassurance to clinicians and third sector service providers that women experiencing intimate partner violence benefit as much as women not reporting intimate partner violence from evidence-based psychological interventions for common mental disorders, when delivered by appropriately trained and supervised practitioners. Future research should investigate whether adapting evidence-based psychological interventions for common mental disorders to address intimate partner violence enhances their acceptability, feasibility, and effectiveness in LMICs.

A major review^11^ of evidence and research priorities for psychological treatments emphasised the need for high-quality randomised controlled trials (RCTs) investigating their scale-up to meet worldwide need, and personalisation to address complexity. For LMICs, the authors advocated brief, streamlined, and locally adapted therapies delivered by less specialised staff (task sharing). However, most published reports of such interventions do not analyse sex and gender differences or the effects of gendered risk factors on outcomes.^12^ Indeed, intimate partner violence is rarely recorded by trials of mental health interventions,^13^ and evidence is scarce on treatments tailored to address the experiences and meet the needs of women with common mental disorders (CMDs) who are also experiencing intimate partner violence, especially in LMICs.^14^

There is growing evidence for the effectiveness of brief, task-shared psychological interventions for CMDs, such as depression and post-traumatic stress disorder (PTSD), in LMICs; a meta-analysis^15^ of 27 RCTs found a pooled effect size of 0·49 (95% CI 0·36–0·62). However, studies rarely report key moderators of treatment response and often do not have the statistical power to detect them. A study^16^ of behavioural activation in India found that women reporting intimate partner violence had more severe depressive symptoms than women not reporting intimate partner violence at follow-up, although the frequency of reported intimate partner violence decreased after the intervention. A systematic review^17^ of primary care mental health programmes in LMICs highlighted complex service user needs, including social risk factors, as barriers to successful implementation.

We aimed to investigate whether exposure to intimate partner violence reduces intervention effectiveness for CMDs in LMICs. We anticipated that to address CMDs effectively in women experiencing intimate partner violence, psychological interventions should be tailored to address the complexities and sensitivities surrounding symptoms in this context, such as acknowledging distress arising from abuse and the effects of psychological abuse. Since most published psychological interventions in LMICs have not been tailored to address intimate partner violence or fulfil WHO guidelines, we hypothesised that intimate partner violence exposure would reduce their effectiveness for CMDs. We focused this study on evidence from LMICs rather than high income countries because of the higher prevalence in LMICs of intimate partner violence, scarce options for women experiencing intimate partner violence in less-resourced settings and the brief, task-shared psychological intervention models that constitute the majority of available research evidence.

## Methods

### Search strategy and selection criteria

We followed PRISMA guidelines throughout our review. We searched Medline, Embase, PsycInfo, Global Health (via Ovid), 3ie, CINAHL, Cochrane Central, LILACS, PILOTS, SciELO, Scopus, and Web of Science Core Collection databases for eligible records using search terms pertaining to “randomised controlled trials”, “psychological interventions”, “common mental disorders”, and “low-income and middle-income countries”. Full search criteria, including all search terms, are listed in the appendix (pp 1–4). RK first did this search on Feb 12, 2018 and repeated it on Aug 16, 2019. RK did supplementary searches of trials registries, with backward and forward citation tracking of included studies. Only peer-reviewed papers published in academic journals were searched for, given the focus on RCTs. RK imported all references from online databases into Endnote X8. After automatically removing duplicates, RK imported the remaining references into Covidence online, for screening. RK screened titles and abstracts of all references, using a hierarchical approach to exclusions— in the order of design (RCT), intervention (psychological), setting (low-income or middle-income country), population (adolescent or adult females), and indication (CMDs)—before a full-text review of whether current or previous intimate partner violence was measured. An independent reviewer (KS) screened the titles and abstracts of 278 (5%) of 5452 records; full texts were single-screened by RK only. Disagreements about exclusion and implications for other exclusions were resolved through discussion between KS and RK.

We obtained aggregate, subgroup data from eligible records by approaching individual authors. Data comprised number of participants (preintervention and postintervention) and mean scores (SD) of outcome measures for women participants by intimate partner violence exposure (yes or no), in both intervention and control groups. Data extraction by RK was repeated by an independent reviewer (SP). Due to time and resource constraints, a planned individual participant data meta-analysis was deferred to a future study.

Eligible studies were required to include female adolescents (aged 13–17 years) or adults (aged ≥18 years) living in LMICs, according to World Bank criteria at the time of data collection.^18^ Mixed-gender studies were included if study authors provided data disaggregated by sex. Studies of HICs were excluded owing to anticipated heterogeneity in comparison to LMICs, arising from the types of interventions offered and women having greater access to education and services, thus assisting them to respond to intimate partner violence and its mental health consequences.

We used a broad definition of psychological intervention as any talking-based therapeutic treatment delivered with the stated aim of improving the primary outcome of a CMD. We included RCTs of psychological interventions delivered to participants diagnosed with depression, anxiety, PTSD, or psychological distress at baseline, compared with any comparator, including treatment as usual. Primary outcomes of included studies were symptoms of one or more CMD. Any study meeting these criteria was eligible for inclusion only if participant outcomes could be separated into women who did and women who did not report exposure to intimate partner violence (as defined by WHO). Further details of included studies are given in the table. Given our hypothesis, of a moderating effect of intimate partner violence on CMD treatment response, we excluded studies targeting psychotic, cognitive (eg, dementia, traumatic brain injury, or intellectual disability), neurodevelopmental, substance use, and personality disorders. Studies that excluded women not experiencing intimate partner violence were not included in our analysis. No language or date exclusions were applied.

**Table:** Characteristics of included records

	**Setting**	**Population**	**Study design**	**Inclusion measure(s)**	**Exclusion criteria**	**Measures of IPV and CMD**	**Interventions (n for which data were available)**	**Control (n for which data were available)**	**Intervention training and delivery**	**Supervision and quality control**	**Intervention description**
Bass et al (2016)	Dohuk governorate in Kurdistan, northern Iraq	Individuals (aged ≥18 years) referred by clinic doctors and former prisoner organisations	Individual RCT	Scoring ≥20 on locally adapted and validated HSCL-25 depression scale, endorsing two selected symptoms of DSM-IV depression, and having experienced or witnessed physical torture, imprisonment, or military attacks	Current psychotic episode or active suicidality or lacking mental capacity to consent to participate	Experience of any domestic violence (no time limit); HSCL-25 depression scale; HSCL-25 anxiety scale; HTQ PTSD scale	A trauma-informed support, skills, and psychoeducation intervention (n=54)	Waiting list control: CMHWs contacted participants monthly, usually by telephone, to check for substantially greater distress or new risks to themselves or others (n=15)	11 CMHWs attended 2 weeks of training, adopting a social work model of help and support, using locally informed training materials, then attended more advanced and refresher training over 2 years	Weekly check-ins via mobile phone and monthly onsite field supervision in groups led by a psychiatrist; supervising psychiatrist reviewed clinical notes for CMHW responses to client needs and checklists of potential activities that could have been provided	Time-limited, trauma-informed support, skills, and psychoeducation intervention, incorporating response sessions where strategies to address symptoms of depression, anxiety, and grief were taught; 6–12 individual sessions at Ministry of Health clinics
Bolton et el (2014)	Rural areas of Erbil and Sulaimaniyah governorates in Kurdistan, northern Iraq	Individuals (aged ≥18 years) referred by doctors and nurses at 14 participating Ministry of Health primary care clinics, one outpatient clinic	Individual RCT	Scoring ≥20 on locally adapted and validated HSCL-25 depression scale, scoring 2 (often) or 3 (always) for DSM-IV criteria of depressive symptoms or anhedonia, and having experienced or witnessed physical torture, imprisonment, or military attacks	Not fluent in Sorani Kurdish; current psychotic symptoms; active suicidality; lacking mental capacity to consent to participate	Experience of any domestic violence (no time limit); HSCL-25 depression scale; HSCL-25 anxiety scale; HTQ PTSD scale	BATD (n=65); CPT (n=58); both interventions were adapted for this setting	Waiting list control: CMHWs contacted participants monthly to enquire generally about the severity of their symptoms and if they were a danger to themselves or others; BATD (n=16); CPT (n=23)	20 CMHWs: interested local primary care clinical staff (completed high school and some postgraduate or role-specific training) with experience of working in rural areas with survivors of torture and trauma and had attended supportive counselling training; CMHWs were randomly assigned to training BATD or CPT using apprenticeship model; 2 weeks' training delivered by USA-based trainers; further training provided by local supervisors	Local supervisors received remote online weekly training and oversight from USA-based trainers	BATD focuses on strategies to encourage structured engagement in healthy and positive values-based behaviours; CPT includes cognitive restructuring and emotional processing of traumatic events; 12 individual sessions delivered in private spaces provided by clinics
Brown et al (2018), Tol et al (2018), and Tol et al (2019)	14 villages of a refugee settlement in northern Uganda	Women (aged ≥18 years) from South Sudan screened through random household sampling of residents	Superiority cluster RCT, stratified by union council (smallest subdistrict administrative unit)	Psychological distress: Juba Arabic Kessler 6 score of >5	Imminent suicide or life-threatening risk; severe mental disorder; unable to understand study materials; or not able to speak Juba Arabic	Three adapted items from the WHO Violence Against Women measure (past 12 months); PHQ-9; PCL-6; Kessler 6	Guided, locally adapted SH+ based on acceptance and commitment therapy plus EUC; PHQ-9, PCL-6 (n=318); Kessler 6 (n=331)	EUC: 10–15 min home visit from CPA, trained village health team member and refugee, covering psychological distress, strategies for overthinking, and information about sources of support; PHQ-9, PCL-6 (n=360); Kessler 6 (n=363)	Two facilitators (minimum: completed secondary education) with experience of psychosocial activities or community mobilisation who spoke Juba Arabic and English attended two 4-day training stages, the second was led by a WHO master trainer	Written facilitator guide; advice not to give detailed explanations; supervised by social worker, clinical supervisor, and team leader; remotely supervised as needed by WHO master trainer; each facilitator had two competency checks	Present moment awareness skills and grounding, diffusion from and acceptance of difficult thoughts and feelings; identifying valued life directions; taking action; self-directed and other-directed compassion; five 2-h group facilitator-led workshops with audio-recorded materials and accompanying pictorial guide
Bryant et al (2017)	Households in peri-urban areas of Nairobi, Kenya	Adult women residing in every 10th household with a history of gender-based violence	Individual RCT with parallel assignment	GHQ-12 score of >2 and WHODAS (version 2.0) score of >16	Imminent plans of suicide; psychotic disorders; severe cognitive impairment	Any previous or current experience of interpersonal violence on either life events checklist or WHO Violence Against Women Instrument (past 12 months); PCL-5; GHQ-12	5-week course of PM+ (n=209)	EUC: referral to primary care health centre for non-specific counselling by one of six unsupervised nurses (session number, strategies, and rescheduling determined by nurse); nurses had 14 years of education, including a diploma and several years of HIV counselling experience; nurses received 2 days of manualised training and a 1-day Psychological First Aid course (n=208)	23 lay CHWs with 10 years of school education but no previous mental health training or experience received 64 hours of training over 8 days; training included knowledge of common mental health conditions, basic counselling, PM+, self-care strategies, gender-based violence issues, ethics, and confidentiality; CHWs received 1-day Psychological First Aid training on managing people in crisis who need immediate attention and potential referral	Each CHW delivered PM+ to about three clients under local supervision before mock interview competency assessment; three CHWs failed or did not take part; two local experienced psychologists trained in PM+ supervised CHWs for 2 h per week in four groups of five CHWs; supervisors received 1·5 h weekly training and mentorship via Skype; 10% of randomly selected PM+ sessions were observed by supervisor using checklist	Session 1: introduction to PM+, motivational interviewing, psychoeducation, stress management; session 2: problem-solving strategies focused on participant-nominated problems, review; session 3: behavioural activation and review; session 4: strengthening social supports and review; session 5: reinforcement of all strategies and relapse prevention education; five 90-min, weekly individual sessionsdelivered at home unless alternative preferred for safety or privacy reasons; each CHW provided PM+ to 8–12 women
Chibanda et al (2016)	24 primary care clinics in accessible locations of Harare, Zimbabwe which had mobile network coverage, were willing to take part, and had data on stratification variables	Adults (aged ≥18 years) living in the area randomly selected from their queue position number, until 24 participants were enrolled per clinic	Cluster RCT, stratified by HIV status, housing density, clinic size, and sex	SSQ-14 score of >8	Pregnant or ≤3 months' postpartum women; people unable to understand the study in English or Shona; suicidal intent; end-stage AIDS; currently receiving psychiatric care or presenting with acute psychosis; intoxication or dementia	Experience of any domestic upheaval in the past 6 months; PHQ-9; GAD-7; SSQ-14	Friendship bench, a manualised problem-solving therapy intervention done on a bench in a discreet area outside the clinic plus EUC (n=230)	EUC: nurse-led evaluation, brief support counselling, medication, information, education, support on CMDs, assessment for antidepressants prescribed by nurse, and referral to psychiatric facility if needed; 2–3 text messages or calls, including reminder to attend follow-up assessments (n=216)	Female lay health workers (mean age: 53 years, mean education: 10 years) who were able to use a mobile phone and were living in the study area attended 9 days of training	Supervision and support from trained senior health promotion officers; groups facilitated by women who previously attended friendship bench and received basic group management training from study clinicians; all sessions audio-recorded and assessed using a checklist	Participants identified a problem; lay health workers sought more positive orientation towards resolving problems, empowering a greater sense of coping and control over life; session 1: opening the Mind (guided problem identification and action planning), uplifting and strengthening; subsequent sessions built on the first; six individual friendship bench sessions lasting 1 h (session 1), then 30–45 min; up to six text messages or calls; invitation to sixsessions of peer-led group support after four sessions; peer-led group support sharing experience whilst crocheting
Ertl et al (2011)	Anaka, Awer, and Padibe, northern Uganda (selected for varied war exposure)	Former child soldiers meeting DSM-IV criteria for PTSD and screening >15 on the PTSD scale, within a population-based survey of 1113 young people aged 13–25 years	Individual RCT	Confirmed PTSD on CAPS when reviewed; participants with suicidal ideation, substance abuse, or depression were not excluded, to preserve a naturalistic sample	Psychotic symptoms	Items on the Violence, War, and Abduction Exposure Scale (no time limit); MINI; CAPS	NET (n=16); academic catch-up programme with elements of supportive counselling (n=19)	Waiting list control (n=12)	14 (seven female) intensively-trained local lay counsellors	Treatment fidelity and therapeutic competence monitored by supervision case discussions, observation, and evaluation of treatment sessions via video and review of obligatory treatment process notes for each session; NET testimonies were reviewed for trauma focus and richness of detail	Session 1 (both active groups): PTSD psychoeducation, intervention rationale; NET participants made detailed chronological biographies, working to reconstruct fragmented memories of traumatic events and to achieve habituation; academic catch-up: counselling to cope with symptoms and address current problems, alongside academic training; final session: text or exercise books provided; eight individual sessions lasting 90–120 min, scheduled three times per week in participant's home in internally displaced persons' camp
Fuhr et al (2019)	North district of Goa, India	Pregnant women (second or third trimester; aged ≥18 years) attending one of two antenatal clinics and two primary healthcare centres	Individual RCT	PHQ-9 of ≥10	Not intending to remain in the study area for ≥1 year; not speaking Konkani, Hindi or Marathi; needing immediate psychiatric or medical inpatient care	Experience of any domestic violence in the past 3 months; PHQ-9	THPP plus EUC (n=140)	EUC: participants and gynaecologist informed of positive depression screening; gynaecologist received adapted mhGAP treatment guidelines for perinatal depression, including guidance on referring women with severe depression or suicide risk to specialist psychiatric care; participants received information about seeking health care during pregnancy and afterwards (n=140)	26 middle-aged lay women (Sakhi) with at least one child, without mental health training, with expressed interest in helping and supporting women in their community; Sakhi were recruited through word of mouth, especially via women's self-help groups and CHWs, and were selected for good communication skills; Sakhis received 25–40 h of interactive classroom training focused on intervention content and relationship-building skills; training incorporated discussion and role plays	Sakhis delivered 2–4 sessions to more than two women in initial 2-month clinical internship, before role play-based competence assessment; Sakhis received peer-led supervision every 14 days in groups of four to five; 50% attended by supervisor; supervision discussed audio-recorded sessions, rated on Therapy Quality Scale; session quality monitored by independent audio ratings of a random 5% sample	THPP adapted for peer delivery from THP by focusing on behavioural activation; four phases comprised prenatal: 1–6 sessions during second and third trimester, starting within 3 days of recruitment; early Infancy: 1–4 sessions in the 2 months after childbirth; middle Infancy: two sessions 3–4 months after childbirth; and late Infancy: two sessions 5–6 months after childbirth; 6–14 individual sessions lasting 30–45 min delivered over 7–12 months; depending on trimester of recruitment, location was participant's home, unless alternative requested
Grundlingh et al (2017)	Luwero district, near Kampala (both rural and urban)	Ugandan university-qualified research assistants employed to interview children about interpersonal violence by a study	Individual RCT with parallel assignment	Any research assistant employed by the Good Schools Study	None	WHO Multi Country Study items, including physical, sexual, and emotional violence from partners (past 12 months); SRQ-20	Three sessions of weekly Group Debriefings for Secondary Distress, including content from Critical Incident Stress Debriefing (n=15)	Weekly film screenings selected for light-hearted, uplifting content presented as fun and relaxing; participants received the intervention after study completion (n=19)	Debriefings delivered by intervention designer and first author of study, a health-care professional with training and experience of facilitating health promotion activities in small groups	None mentioned	Storytelling, identifying emotional responses to stories, psychoeducation, practical information to normalise reactions to distressing events; session 1: group discussing own study experiences; session 2: connecting current experiences with own life experiences; session 3: discussing societal and community responses to issues raised, focusing on constructively addressing; three 90–120 min face-to-face group sessions at the end of the working day at participants' hotel; each session started with an ice-breaker for relaxed atmosphere and cohesion
Lund et al (2019)	Two antenatal clinics in a low-income township of Cape Town, South Africa	IsiXhosa-speaking pregnant women (aged ≥18 years) at ≤26 weeks' gestation, living in the study area	Individual RCT	EPDS score of >12	Requiring urgent medical attention; having schizophrenia, bipolar mood disorder or current psychotic episode; unable to give informed consent	Experience of physical or sexual violence by a partner in the past 3 months; HDRS	Structured, manualised basic counselling from one of six trained CHWs (n=205)	EUC: Three monthly phone calls from one of two CHWs not trained in basic counselling; brief conversation about emotions, major life changes, sources of support, community perinatal services, and suicidal ideation (n=214)	12 CHWs employed by local NGO attended 5 days of basic counselling and intervention training delivered by a trained clinical social worker (clinical social worker); of these, six were chosen to deliver intervention based on motivation, understanding, empathy, and interpersonal style; refresher training available if needed	Weekly group mental health support and supervision from specialist social worker: case reviews, discussing difficult cases, developing supportive relationships with health providers, managing emergencies; monthly individual supervision discussing progress; initial session observed by specialist counsellor, all sessions audio recorded; random specialist review; CHW checklist for each session	Aspects of psychoeducation, problem solving therapy, behavioural activation, cognitive reframing (healthy thinking), and relaxation training; six sessions of basic counselling lasting 1 h each over 3–4 months, with follow-up phone calls if sessions missed; sessions delivered every 2 weeks in clinic or participant's home, aligned with routine antenatal care where possible
Patel et al (2017) and Weobong et al (2017)	Ten primary health centres in Goa, India	Adults (aged 18–65 years)	Individual RCT, stratified by primary health centre and sex	PHQ-9 score of >14	Pregnant women; requiring urgent medical attention; unable to communicate clearly	Experience of physical or psychological IPV (no time limit); PHQ-9	HAP, a manualised psychological treatment plus EUC (n=103)	EUC: screening results provided to participant and physician and mhGAP manual provided, including information about referral for psychiatric care (n=116)	Lay counsellors attended 3 weeks of participatory workshops covering HAP and CAP followed by a 6-month internship phase of delivery with peer-led group supervision	Five local specialists (trained and supervised by international expert in behavioural activation) trained and supervised 11 lay counsellors who passed competency assessment; randomly selected 10% recorded sessions rated on HAP therapy quality scale and twice monthly individual supervision, plus weekly peer-led supervision in groups of four to six; random supervisor quality review of recorded recruitment interviews; random independent expert assessment of 10% of all sessions	Based on behavioural activation, including psychoeducation, behavioural assessment, activity monitoring, activity structuring and scheduling, activating social networks, and problem solving, with additional behavioural strategies for communication skills, sleep and relaxation; 6–8 individual sessions lasting 30–40 min, extended from 6–8 sessions if consistently high PHQ-9 and absent activation; sessions delivered at primary health centre or participant's home; by telephone when needed
Sikander et al (2018)	Ten randomly assigned village clusters in a rural subdistrict of Rawalpindi, Pakistan	Pregnant women (aged ≥18 years) in their third trimester who were registered for village-based health care from LHWs living in the study setting	Cluster RCT	Urdu PHQ-9 score of >10	Not intending to reside in the study setting for ≥1 year; not speaking Urdu, Punjabi or Potohari; needing immediate medical or psychiatric inpatient care	Experience of any domestic violence in the past 3 months; PHQ-9	THPP plus EUC (n=275)	EUC: LHWs informed of positive depression screening and provided with mhGAP guidelines for perinatal depression; participants given information on how to seek help (n=282)	Three LHWs per village cluster: volunteer peers (married women with children, aged 30–35 years), selected for good communication skills, received brief classroom training and regular group training	Field supervision by local non-specialist THPP trainers supervised by a specialist therapist; ENACT-based ratings of competency	Behavioural activation; narratives and pictures challenging unhelpful thinking and behaviour; ten individual at-home sessions and four group sessions at LHW's home lasting 30–45 min; front-loaded so more frequent during pregnancy
Steinert et al (2017)	Mekong Project sites in Phnom Penh City and nearby Kandal Province, Cambodia	Adults (aged ≥18 years) seeking help from the Mekong Project, which provided free psychological help to traumatised civilians	Individual RCT	PCL-C score of ≥44	Comorbid psychosis; organic brain disorder; cognitive impairment; dementia; acute suicidality; acute treatment need; severe communication difficulties; ongoing therapy in past 2 years	Asked type of trauma for which therapy was sought, including domestic violence (no time limit); HSCL-25 depression scale; HSCL-25 anxiety scale; HTQ PTSD scale; HSCL-25 total score	Resource-oriented trauma therapy plus EMDR resource installation (ROTATE; n=34)	Waiting list control (n=17)	Six local therapists, each with a Masters degree in psychology from Royal University of Phnom Penh, completed a 3 year course in trauma therapy led by an experienced therapist	Not mentioned	Manualised treatment focused on resource activation to enhance emotion regulation, taught grounding techniques and applied EMDR technique of resource development and installation; 5 h in weekly individual sessions
Latif et al (2017)	Urban Karachi, Pakistan	Literate women (aged 18–40 years) attending one shelter home and three NGOs	Individual RCT	Evidence of depression, anxiety, and domestic violence on screening	Depression with psychotic features; bipolar affective disorder; cognitive impairment; physical illness	HITS scale; Aga Khan Anxiety and Depression Scale (Urdu Version)	Group CBT with a helper to guide homework (n=100)	Self-help manual with a helper to guide reading in groups of ten (n=100)	Delivered by a therapist; details not specified	Not mentioned	Psychoeducation on depression and anxiety, coping skills for anxiety, activity scheduling, nutrition, physical activity, problem-solving, linking situations, thoughts and emotions, cognitive restructuring, core beliefs, assertiveness training, communication skills; ten sessions in twice-weekly groups of ten women, lasting 90 min
Orang et al (2018)	Urban Tehran, Iran	Women (aged 16–60 years) living with IPV in contact with health professionals, social activists, or other staff working with women experiencing abuse	Individual RCT	PSS-I score of ≥15; experienced IPV in the past year; married or living with a violent partner at the time of interview	Substance abuse; schizophrenia; epilepsy; intellectual disability	Persian-translated versions of CAS, PSS-I, and PHQ-9	NET plus psychoeducation (n=17)	TAU: individually-tailored life skill training and supportive counselling, including joint sessions with abusive husbands, CBT, acceptance and commitment therapy; focused on currently important events; psychoeducation: normalisation, legitimisation and description of trauma reactions; sessions lasted 90-120 min (n=17)	Control group delivered by three local female Masters-qualified psychology graduates; intervention delivered by two of these three, trained in NET in workshops delivered by experienced NET trainers; first sessions of NET were supervised by clinical psychologist with NET expertise and a local NET expert to ensure that sessions followed NET manual guidelines	Assessments at baseline and months 3 and 6 by independent female Masters-qualified psychology graduates who were masked to treatment allocation; supervised by a doctoral-level clinical psychologist	Participants shaped their traumatic life experience into a written narrative of their life; cognitive restructuring to deconstruct violence-related traumatic events, ending with focus on future expectations, goals, worries and hopes; up to 15 NET sessions lasting 120–150 min each

Details on the timing and safety of the included records are listed in the appendix (pp 5–7). Individual RCT refers to studies in which participants were randomly assigned to therapy at the individual level, as opposed to at the level of the health-care setting (ie, cluster RCT). BATD=Behavioural Activation Treatment for Depression. CAP=Counselling for Alcohol Problems. CAPS=clinician-administered PTSD Scale. CAS=Composite Abuse Scale. CMD=common mental disorder. CHW=community health worker. CMHW=community mental health worker. CPA=community psychosocial assistant. CPT=cognitive processing therapy. DSM=Diagnostic and Statistical Manual of Mental Disorders. EMDR=eye movement desensitisation reprogramming. EPDS= Edinburgh Postnatal Depression Scale. ENACT=Enhancing Assessment of Common Therapeutic factors assessment. EUC=enhanced usual care. GAD-7=Generalised Anxiety Disorder-7 Assessment. GHQ-12=General Health Questionnaire-12. HAP=Healthy Activity Programme. HSCL=Hopkins Symptom Checklist. HTQ=Harvard Trauma Questionnaire. IPV=intimate partner violence. LHW=lady health worker. mhGAP=WHO Mental Health Gap Action Programme. MINI=Mini-International Neuropsychiatric Interview. NET=narrative exposure therapy. NGO=non-governmental organisation. PCL=PTSD checklist. PCL-C=PTSD checklist–civilian version. PHQ-9=Patient Health Questionnaire 9. PM+=Problem Management Plus. PSS-I=Post-traumatic Stress Symptom scale–Interview. PTSD=Post-Traumatic Stress Disorder. RCT=randomised controlled trial. ROTATE=Resource-Oriented Trauma therapy and EMDR resource installation. SH+=Self-Help Plus. SRQ-20=Self-Reporting Questionnaire-20. SSQ-14=Shona Symptom Questionnaire. TAU=treatment as usual. THPP=Thinking Healthy Programme-Peers. WHODAS=WHO Disability Assessment Schedule..

Owing to the likelihood of intimate partner violence being measured but not mentioned in titles and abstracts, we first searched for studies meeting all but this criterion (appendix pp 1–4) before doing a full-text review to determine whether intimate partner violence exposure was measured.

As a systematic review and meta-analysis, ethical approval was not sought for this study. The individual studies included in the analyses obtained ethical approval independently.

### Data analysis

We did independent samples t-tests to compare mean baseline CMD symptom scores between groups reporting and not reporting intimate partner violence in each included study. We did random-effects meta-analyses using Stata (version 15; College Station, TX, USA) for any CMD outcome measured by at least four studies,^19^ estimating heterogeneity using *I*^2^ and visually inspecting the funnel plot for meta-analyses including at least ten studies. Because within-study group differences were provided, we first separately calculated the standardised mean difference (SMD) and SE in treatment effect between participants with and without intimate partner violence exposure in each intervention and control group, before a second random-effects meta-analysis of the difference between SMDs (dSMD) between intervention and control groups. RK extracted study design and implementation details using a piloted table and evaluated all included studies using the Cochrane risk of bias tool.^20^ We anticipated that, in settings where intimate partner violence was sufficiently prevalent to be measured, female therapists might have been considered more culturally acceptable to female participants. We did post-hoc subgroup analyses to compare dSMDs of trauma-focused interventions versus generic psychological interventions, female-delivered interventions versus mixed gender-delivered interventions, novel treatments for LMICs versus those with an established evidence base in high-income countries, and those asking only about recent (within the past 12 months) intimate partner violence versus lifetime intimate partner violence. We did sensitivity analyses by reviewing changes to pooled dSMD estimates when one study was removed from each meta-analysis at a time.

This study is registered on PROSPERO, number CRD42017078611.

### Role of the funding source

The funders of the study had no role in study design, data collection, data analysis, data interpretation, or writing of the report. The corresponding author had full access to all the data in the study and had final responsibility for the decision to submit for publication.

## Results

**Figure 1:**
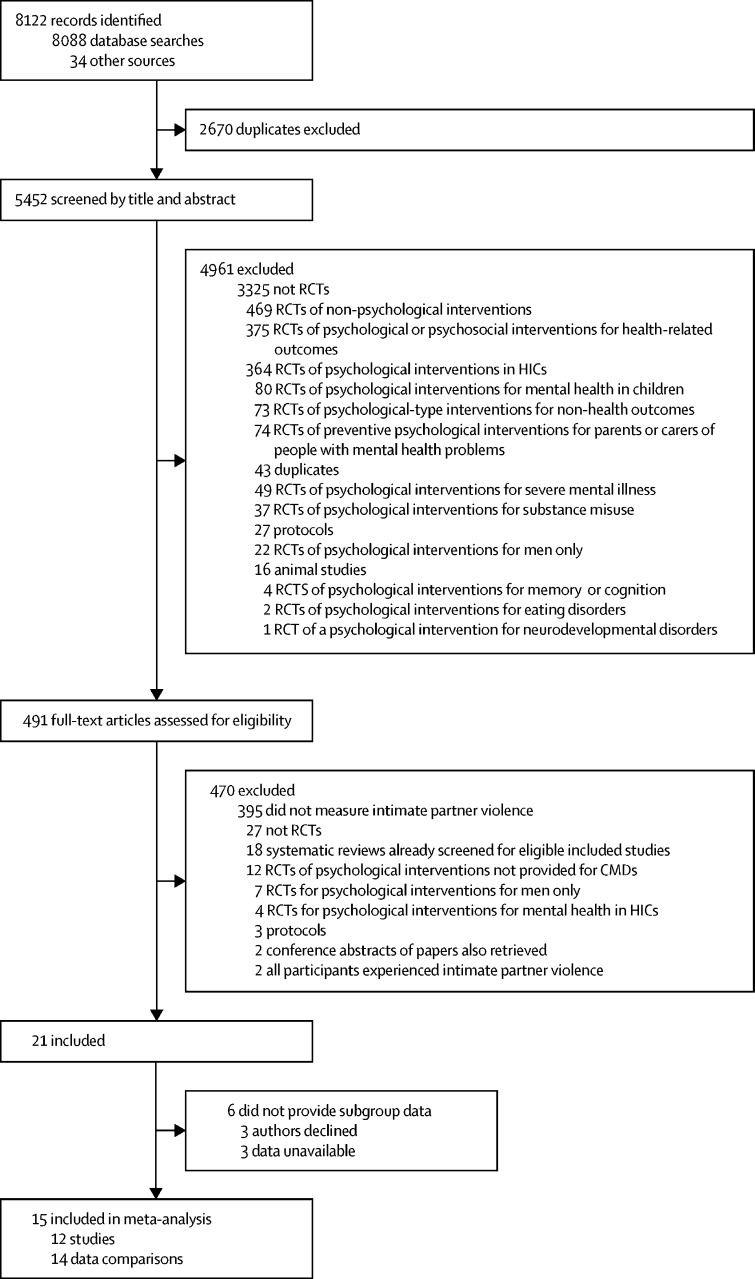
Study selection We followed PRISMA guidelines (appendix pp 17–18). Two of the 15 records were three-group studies and thus provided two data comparisons for meta-analysis. Three of the 15 records reported one of the already included 12 studies. RCT=randomised controlled trial. HIC=high-income country. CMD=common mental disorder.

Our search identified 8088 records and a further 34 were identified by manual screening of citations (figure 1). After excluding 2670 duplicates, we screened the titles and abstracts of 5452 records, of which 4961 were excluded. We screened the full texts of the remaining 491 records, yielding 21 eligible records. Data were unavailable for six of the 21 eligible records; authors of 15 records shared data for meta-analysis pertaining to 12 studies. Screening decision agreement was 98·6% (274 of 278 records); all disagreements were resolved through discussion and reasons for differences were established to inform subsequent decisions. Of 470 full-text records excluded, 395 (84%) were RCTs of psychological interventions in LMICs which did not measure intimate partner violence exposure. Other reasons for exclusion are listed in figure 1.

The 12 included studies described 14 interventions (two studies reported three-group trials,^21^, ^22^ enabling meta-analysis of four CMD symptom groups (anxiety, PTSD, depression, and psychological distress). We excluded two studies^23^, ^24^ from our meta-analysis because all participants reported intimate partner violence (table). Seven (50%) of 14 interventions included in our meta-analysis were implemented in African countries (one each from Kenya, South Africa, and Zimbabwe, and four from Uganda) and seven (50%) of 14 in Asian countries (one each from Cambodia and Pakistan, two from India, and three from Iraq). Four (29%) of 14 studies took place in post-conflict regions, one (7%) in a refugee camp, and the remaining seven (50%) in regions unaffected by recent conflict (defined as conflict within the 5 years before data collection). All studies were published after 2010 and used individual randomisation of participants, except three studies^25^, ^26^, ^27^ in which randomisation was at the cluster level. No studies included participants younger than 13 years.

Studies measured postintervention outcomes at variable times: immediately,^28^, ^29^, ^30^ 3 months^22^, ^27^, ^31^, ^32^ or 6 months^21^, ^25^ post-participation, and 3 months^33^ or 6 months^26^, ^34^ postpartum. Studies varied in their use of validated intimate partner violence measures, including items from the WHO Violence Against Women Instrument^27^, ^28^, ^31^ or Violence, War, Abduction and Exposure Scale.^22^ Most studies asked a single question about intimate partner violence^32^, ^33^ or domestic violence^21^, ^26^, ^29^, ^30^, ^34^ exposure, which might have included abuse by non-partners. Some studies^26^, ^33^, ^34^ asked about intimate partner violence in the past 3 months only. One study^25^ asked about domestic upheaval in the past 6 months. One study^32^ measured intimate partner violence exposure at 3-month follow-up.

A higher than anticipated number of included studies evaluated interventions featuring components explicitly designed to address traumatic experiences (four reports) and a lower than anticipated number were delivered by female therapists (five reports); no study described matching participants and therapists by sex.

Included interventions were delivered by various staff: a public health professional and researcher,^28^ psychology Masters-educated therapists,^29^ community mental health workers,^21^, ^30^ general health workers,^31^, ^33^ other community workers,^27^ and lay individuals.^22^, ^25^, ^26^, ^32^, ^34^ Five studies^25^, ^26^, ^27^, ^32^, ^34^ compared the control intervention (enhanced usual care [EUC]) with the intervention plus EUC, but the content of EUC, and other studies' control interventions, varied from face-to-face or telephone-delivered basic counselling^31^, ^33^ to diagnosis and the opportunity for WHO mental health gap action programme intervention guided treatment,^26^, ^32^, ^34^ a home visit,^27^ film screenings,^28^ and waiting lists.^21^, ^22^, ^29^, ^30^ Numbers of sessions ranged from three^28^ to 14,^33^ delivered at clinics,^21^, ^30^ in community settings,^25^, ^27^, ^28^ at home,^22^, ^31^, ^34^ or a mixture of locations.^26^, ^32^, ^33^ Most interventions were delivered individually, with two in groups^27^, ^28^ and one mixed.^26^ All studies, except two,^28^, ^33^ reported significant improvements in at least one CMD in the intervention group compared with the control group. Mean baseline CMD scores differed between women who did and did not report intimate partner violence in none of the five interventions reporting anxiety symptoms, in two (25%) of eight interventions reporting PTSD symptoms, in five (42%) of 12 interventions reporting depression symptoms, and in one (25%) of four interventions reporting psychological distress symptoms (appendix pp 8–9).

**Figure 2:**
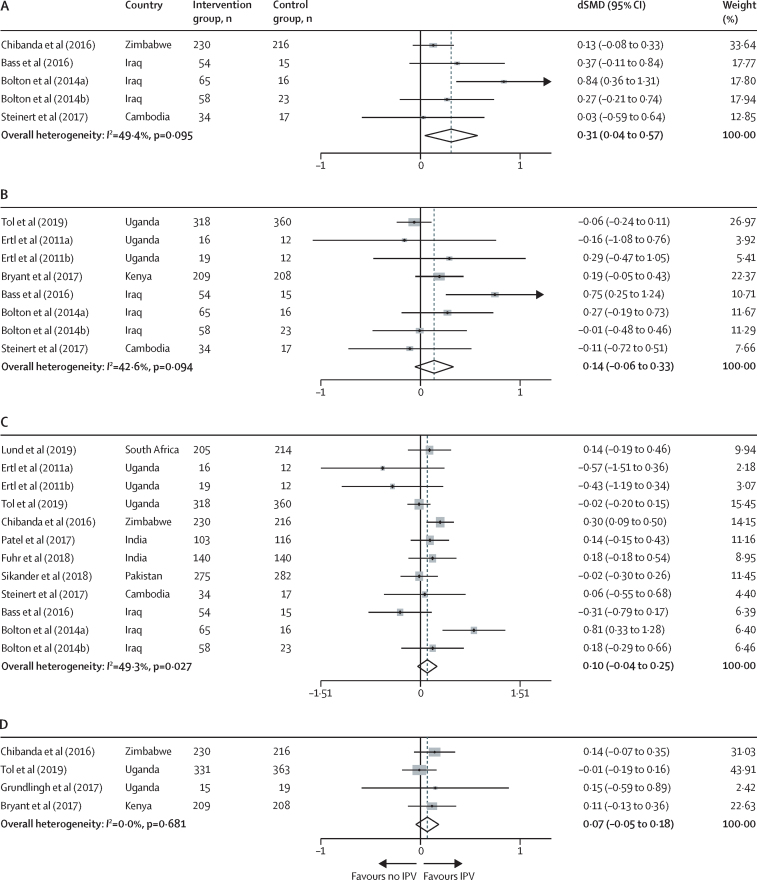
Random-effects meta-analyses of the difference in psychological intervention study effect sizes (via SMD) between women who did and women who did not report exposure to IPV Data are for women with anxiety (A), PTSD (B), depression (C), and psychological distress (D) symptoms. dSMD=difference in standardised mean differences. IPV=intimate partner violence.

Meta-analysis of five interventions measuring preintervention and postintervention anxiety symptoms (728 participants), comprising one large study of problem-solving therapy and four smaller studies of trauma-focused interventions, showed greater reductions in anxiety symptoms among women who reported intimate partner violence exposure than those who did not (dSMD 0·31, 95% CI 0·04–0·57, *I*^2^=49·4%; figure 2A). Meta-analysis of eight interventions measuring preintervention and postintervention PTSD symptoms (1436 participants) showed no difference in PTSD symptom reduction among women who reported intimate partner violence exposure and those who did not (0·14, −0·06 to 0·33, *I*^2^=42·6%; figure 2B). Meta-analysis of 12 interventions measuring preintervention and postintervention depression symptoms (2940 participants) found no difference in depression symptom reduction among women who reported intimate partner violence exposure than those who did not (0·10, −0·04 to 0·25, *I*^2^=49·3%; figure 2C). Meta-analysis of four interventions measuring preintervention and postintervention psychological distress symptoms (1591 participants) found no difference in psychological distress symptoms among women who reported intimate partner violence exposure than those who did not (0·07, −0·05 to 0·18, *I*^2^=0·0%, p=0·681; figure 2D).

Cochrane risk of bias assessments (appendix p 10) showed moderate risk of bias in eight (67%) of 12 studies and low risk in four (33%). All 12 studies were unable to mask participants and professionals to the psychological or control intervention which they received or delivered. Only PTSD symptom results comprised solely moderate-risk studies. Only the depression meta-analysis included more than ten studies. The funnel plot for studies included in the depression meta-analysis was asymmetrical, suggesting potential publication bias (appendix p 11).

Prespecified subgroup analyses by therapist role (specialist *vs* layperson), format (group *vs* individual), and context (stand-alone *vs* embedded in a wider programme) were not done owing to low study numbers in one or more subgroups.

We did post-hoc subgroup comparisons to explore whether the dSMD between women who did and did not disclose intimate partner violence was driven by key intervention design features. Comparing explicitly trauma-focused interventions with more generic behavioural activation and cognitive behavioural therapy-focused interventions did not show differences in the moderation effect of intimate partner violence (appendix pp 12–13).

**Figure 3:**
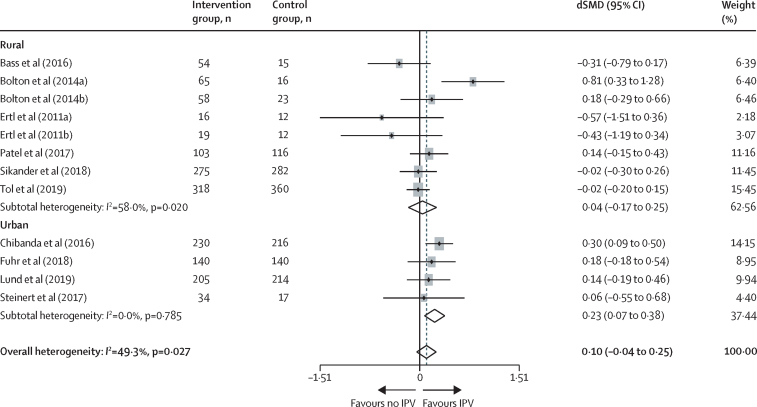
Random-effects meta-analyses of the difference in psychological intervention study effect sizes (via SMD) for depression symptoms between women who did and women who did not report exposure to IPV, by residential setting dSMD=difference in standardised mean differences. IPV=intimate partner violence.

Subgroup comparisons between interventions done in rural and urban locations showed no difference in depression symptoms in women reporting intimate partner violence relative to women not reporting intimate partner violence in urban (dSMD 0·23, 95% CI 0·07 to 0·38) versus rural (0·04, −0·17 to 0·25) locations (figure 3).

**Figure 4:**
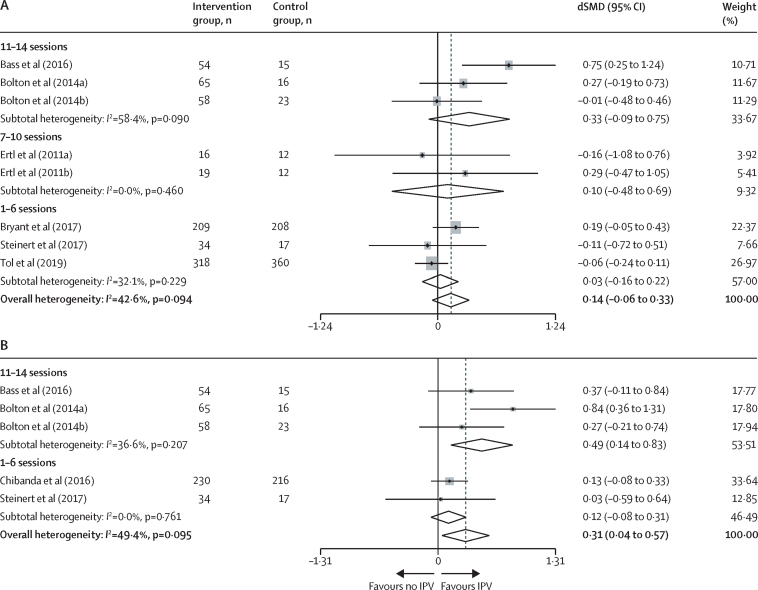
Random-effects meta-analyses of the difference in psychological intervention study effect sizes (via SMD) between women who did and women who did not report exposure to IPV, by number of treatment sessions Data are for women with PTSD (A) and anxiety (B) symptoms. The difference in anxiety symptoms was affected by location (more sessions offered in rural locations) and exposure to conflict (more sessions offered to conflict-exposed populations). For anxiety symptoms, none of the studies offered 7–10 sessions. dSMD=difference in standardised mean differences. IPV=intimate partner violence. PTSD=post-traumatic stress disorder.

Stratifying interventions by the maximum number of sessions offered showed no difference in PTSD symptoms in women reporting intimate partner violence relative to women not reporting intimate partner violence (1–6 sessions dSMD 0·03, 95% CI −0·16 to 0·22 *vs* 7–10 sessions 0·10, −0·48 to 0·69 *vs* 11–14 sessions 0·33, −0·09 to 0·75; figure 4A). Similarly, subgroup comparisons showed no association of session number with anxiety symptoms in women reporting intimate partner violence relative to those who did not (1–6 sessions dSMD 0·12, 95% CI −0·08 to 0·31; 11–14 sessions 0·49, 0·14 to 0·83; figure 4B). This association might be confounded by the fact that studies measuring anxiety symptoms and offering 1–6 sessions also took place in urban settings unaffected by recent conflict and studies offering 11–14 sessions took place in rural settings with populations affected by recent conflict.

Our post-hoc comparison of interventions delivered by female only versus mixed gender staff, those asking only about recent versus lifetime intimate partner violence, and novel treatments for LMICs versus those with an established evidence base from HICs did not influence the moderating effect of intimate partner violence (data not shown). Sensitivity analyses showed that removing any one study from each meta-analysis did not alter the pooled dSMD estimate (appendix pp 14–15).

## Discussion

By contrast to our hypothesis, this systematic review and meta-analysis found that women disclosing intimate partner violence benefitted more from psychological interventions for anxiety than women who did not but there were no differences for PTSD, depression, and psychological distress. Despite the well established bidirectional relationship between intimate partner violence and mental health, our findings are the first suggestion of a moderating effect of intimate partner violence exposure on treatment effectiveness, using unpublished subgroup data from 15 (71%) of 21 eligible studies.

We had predicted that the additional trauma of intimate partner violence exposure would reduce women's ability to benefit from psychological interventions for CMDs in LMICs. None of the included interventions specifically addressed intimate partner violence or CMD symptoms arising from intimate partner violence. However, several were designed to be trauma-informed, by addressing cognitive processing of traumatic events,^21^ habituating traumatic memories,^22^ focusing on traumatic experiences,^29^ or debriefing.^28^ The remaining interventions had a more practical focus and were likely to benefit women disclosing intimate partner violence through problem-solving,^25^, ^31^ behavioural activation,^21^, ^26^, ^32^, ^34^ coping skills,^27^, ^30^ or a mixture of methods.^33^

Given that anxiety is an important manifestation of trauma, more trauma-focused interventions might yield even greater gains than generic interventions in women experiencing intimate partner violence. Subgroup analyses did not show differences between intimate partner violence's moderating effect on trauma-focused intervention versus more generic psychological intervention outcomes. This finding might have resulted from the small sample sizes of trauma-focused intervention studies, with restricted power to detect a subgroup difference. However, the two included studies with three-group designs found that narrative exposure therapy was more effective for PTSD in Ugandan former child soldiers than supportive counselling or waiting list^22^ and that behavioural activation was more effective for depression in Iraqi survivors of systematic violence than cognitive processing therapy or waiting list.^21^ More widespread measurement of depression, generalised anxiety, and PTSD symptoms by RCTs comparing different psychological intervention models would enable subgroup differences in treatment response to be tested systematically. Although RCTs done in HICs have reported improvements in CMDs in women experiencing intimate partner violence after cognitive behavioural therapy-informed, mind–body, and trauma-focused psychotherapeutic interventions,^35^ to our knowledge, no RCTs have directly compared generic and trauma-focused approaches.

Unlike other past traumas, intimate partner violence is likely to be an active and continuing stressor, even when the relationship has ended; indeed, estrangement and leaving for a new partner are risk factors for intimate partner femicide.^36^ Problem solving, behavioural activation, cognitive behavioural therapy, and strategies to manage trauma symptoms might have been more effective in women disclosing intimate partner violence because they could be applied to that specific and continuing stressor, affording a sense of mastery or control.^37^

A meta-analysis of RCTs of psychological treatments for CMDs in LMICs found that the most common specific intervention elements were identifying social support, problem-solving, identifying or eliciting affect (or both), linking affect to events, and identifying thoughts.^15^ The most common non-specific elements were empathy, collaboration, active listening, normalising treatment or symptoms (or both), and involving significant others. These were common features of interventions included in this meta-analysis and, again, might have been more effective in treating anxiety symptoms in women able to apply them to the concrete stressor of intimate partner violence.

Studies included in our meta-analysis targeted populations exposed to other traumatic experiences: survivors of torture^30^ and systematic violence^21^ in Iraq, South Sudanese refugees,^27^ Ugandan former child soldiers,^22^ and participants experiencing wider gender-based trauma in Kenya^31^ or trauma in Cambodia.^29^ The remaining studies, whilst not specifically recruiting traumatised participants, were done in settings affected by deprivation, poverty, crime, or HIV infection, where the prevalence of adverse life events is likely to have been high. It is possible that, within these populations, women who felt able to disclose intimate partner violence exposure differed from women who did not in their capacity to benefit from psychological interventions and apply techniques learned during therapy sessions to their daily lives.

The proportions of participants reporting intimate partner violence in included studies were higher than national prevalence estimates (appendix p 16), but since national prevalence estimates are not available for subgroups of women with CMDs, under-detection of intimate partner violence cannot be excluded. Studies that asked a single question about intimate partner violence or asked general questions about relationships rather than specific behaviours are more likely to have underestimated forms of intimate partner violence that require more nuanced discussion, such as psychological abuse, coercive control, and physical and sexual violence. This approach might have led to underestimation of a moderating effect of intimate partner violence. Since not all women experiencing intimate partner violence can be expected to have disclosed it to researchers, those women who did disclose intimate partner violence are unlikely to be a representative sample. Severe coercive control and fear of abuse are likely to have prevented some women from disclosing intimate partner violence and participating in RCTs. Furthermore, women experiencing the most severe intimate partner violence might be the least likely to access the routine health-care settings in which most included studies recruited participants. Our results might, therefore, not be generalisable to the population of women experiencing intimate partner violence, or the subgroup for whom intimate partner violence is most severe. Future studies should quantify the type and severity of intimate partner violence and actively recruit participants through diverse means, including approachable community networks. If included studies did underestimate intimate partner violence, our results could reflect the fact that women who felt able to disclose intimate partner violence were at more advanced stages of psychosocial readiness^38^ in a stages of change model, which could have mediated the potential of their anxiety symptoms to benefit from treatment. Since such mediators were not recorded by included studies, this area is an important focus for future research.

This analysis has a few limitations. Data could not be retrieved from six studies, limiting the completeness of our analysis. 395 otherwise eligible studies were excluded because intimate partner violence was not measured, despite WHO guidelines stating that all women with mental health problems should be asked about intimate partner violence in a safe environment by trained staff. As a result, only the depression meta-analysis included more than ten studies. The inherent heterogeneity of included interventions, study contexts, and evaluation designs restricts the interpretation of our results, but previous meta-analyses of psychological interventions in LMICs^15^ have shown consistent effects despite this variation.

As data were not collected on other moderator variables, we cannot rule out the possibility that intimate partner violence is correlated with another, causative moderator variable. The range of ways that intimate partner violence was measured also contributes statistical variance to these results. However, applying the Checklist for the Appraisal of Moderators and Predictors^39^ to our moderator analyses showed that intimate partner violence is a plausible moderator, which we prespecified and was measured pre-allocation in all but one study.^32^ We tested intimate partner violence as a single candidate moderator, with adequate sample sizes, and presented all results. The effect was consistent across related outcomes and settings and study populations were ecologically valid. Although the moderator effect was not homogeneous across studies, this might result from heterogeneity of study designs, interventions, target groups, and contexts, which was moderate.

Although all included studies were rated at low or moderate risk of bias, differences in research methods used by included studies restricted their comparability. For example, studies measured postintervention outcomes at variable times (immediately,^28^, ^29^, ^30^ 3 months,^22^, ^27^, ^31^, ^32^ or 6 months^21^, ^25^ post-participation, and 3 months^33^ or 6 months^26^, ^34^ postpartum) and varied in their use of validated intimate partner violence measures. Most studies asked a single question about intimate partner violence^32^, ^33^ or domestic violence^21^, ^26^, ^29^, ^30^, ^34^ exposure, which might have included abuse by non-partners. Some studies^26^, ^33^, ^34^ asked about intimate partner violence in the past 3 months only, perhaps underestimating intimate partner violence prevalence and one study^25^ asked about domestic upheaval in the past 6 months, perhaps overestimating intimate partner violence prevalence. One study^32^ measured intimate partner violence exposure at 3-month follow-up.

Studies did not distinguish between ongoing intimate partner violence and intimate partner violence that had ended. Specifying exposure to past only, current only, and both types of intimate partner violence would enhance interpretation, since studies in HIC suggest such violence has cumulative effects on women's mental health.^40^ Many studies incorporated safety protocols to manage adverse mental health events, but none described procedures for responding to intimate partner violence-related risks arising during participation. Future research should apply comprehensive international guidance addressing the ethics, design, and safety of research with women experiencing intimate partner violence.^41^

Our results show the importance of RCTs measuring^13^ and analysing the moderating effects of gendered risk factors, such as intimate partner violence,^12^ on treatment effectiveness, and the benefits of data sharing for analysing group effects with relatively small sample sizes. Women experiencing intimate partner violence face a range of personal, clinician, and health system barriers to accessing care for their mental health.^42^ This study suggests that, where resources are unavailable to tailor psychological interventions for CMDs to the complex needs of women experiencing intimate partner violence, they might benefit as much as women not experiencing intimate partner violence from more generic interventions. Clinical staff require training on asking about and responding to intimate partner violence to address it safely. RCTs of psychological interventions should measure evidence-based moderators of treatment effectiveness, such as intimate partner violence exposure, using validated metrics as part of a minimum reported dataset.

Our systematic review identified just two RCTs of psychological interventions for CMDs tailored for women experiencing intimate partner violence in LMICs, which were excluded from our analyses because all participants reported intimate partner violence. Ten sessions of intimate partner violence-adapted group cognitive behavioural therapy were associated with reduced depression and anxiety severity compared with cognitive behavioural therapy-based self-help groups in Karachi, Pakistan.^23^ Receipt of 10–12 sessions of intimate partner violence-tailored narrative exposure therapy was associated with reduced severity of PTSD and depression at months 3 and 6 of follow-up, compared with treatment as usual (life skills training and supportive counselling) in Tehran, Iran.^24^ These studies support the potential benefits of adapting psychological interventions to meet the complex needs of women experiencing intimate partner violence, which requires further exploration.

The prioritisation of intimate partner violence and its health impacts by international organisations^1^, ^2^ should now be matched by the mental health research community and its funders, focusing on the nature of intimate partner violence's moderating effect on treatment response for anxiety, mediators of effective psychological interventions for women experiencing intimate partner violence, and factors affecting their successful implementation in practice.
