# Supplementary material for: Psychological interventions for common mental disorders in women experiencing intimate partner violence in low-income and middle-income countries: a systematic review and meta-analysis
Source: Lancet Psychiatry. 2020 Feb;7(2):173–90. doi: 10.1016/S2215-0366(19)30510-3 (PMC7029417; doi:10.1016/S2215-0366(19)30510-3)
Supplement: Supplementary appendix [file mmc1.pdf]

THE LANCET
Psychiatry

Supplementary appendix

This appendix formed part of the original submission and has been peer reviewed. We post it as supplied by the authors.

Supplement to: Keynejad RC, Hanlon C, Howard LM. Psychological interventions for common mental disorders in women experiencing intimate partner violence in low-income and middle-income countries: a systematic review and meta-analysis. *Lancet Psychiatry* 2020; **7:** 173–90.

Appendix: Table of Contents

1: Search terms applied in Medline search 1

2: Independent samples t-tests for the difference between baseline mean CMD scores for included studies in meta-analyses of anxiety (A), PTSD (B), depression (C) and psychological distress (D) symptoms 8

3: Cochrane Risk of Bias Tool assessments 10

4: Funnel Plot for studies included in the meta-analysis of depression symptoms 11

5: Random-effects meta-analyses of the difference in psychological intervention study effect sizes (dSMD) between women who did and women who did not report exposure to intimate partner violence (IPV), for anxiety (A), depression (B), PTSD (C) and psychological distress (D) symptoms, comparing generic and explicitly trauma focused interventions 12

6: Sensitivity analyses reviewing changes to pooled dSMD estimates when one study was removed from each meta-analysis at a time, for anxiety (A), PTSD (B), depression (C) and psychological distress (D) symptoms 14

7: Comparison between country(1) or regional IPV prevalence(2) and study prevalence or incidence 16

8: PRISMA 2009 Checklist 17

1: Search terms applied in Medline search

1	exp psychotherapy/ or exp psychotherapy, brief/ or psychotherapy, group/ or psychotherapy, multiple/ or psychotherapy, psychodynamic/
2	(psychosocial or psycho-social).ti,ab,kf,hw.
3	psychotherap*.ti,ab,kf,hw.
4	((psychosocial or psycho-social or psycholog$ or behavio?r* or cognitive) adj3 (intervent$ or therap$ or treat$ or manag$)).ti,ab,kf.
5	talking therap*.ti,ab,kf.
6	problem sol*.ti,ab,kf,sh.
7	talking therapy.mp.
8	exp *Family Therapy/
9	((psychosocial or psycho-social or psycholog$ or behavio?r* or cognitive) adj3 (intervent$ or therap$ or treat$ or manag$)).tw.
10	((acceptance and commitment) or activity scheduling or analytical therap$ or art therap$ or aversion therap$ or balint group or behavio?r activation or behavio$ contrac$ or behavio?r modification or behavio?r therap$ or bibliotherap$ or biofeedback or body therap$ or brief therapy or client cent$ therapy or cognitive behavio?r therap$ or cognitive therap$ or CBT or cCBT or iCBT or cognitive behavio?ral stress management or cognitive restructur$ or colo?r therap$ or compassion focus$ or compassionate therap$ or contingency management or conversion therap$ or conversational therap$ or couples therap$).mp.
11	(((dance therap$ or dialectic$) adj2 therap$) or diffusion therap$ or distraction therap$ or (dream$ adj3 analys$) or eclectic therap$ or emotion$ focus$ therap$ or emotional freedom technique or encounter group therap$ or existential or experiential or exposure therap$ or expressive therap$ or eye movement desensiti#ation or family therap$ or feminist therap$ or focus oriented or free association or freudian or functional analysis or gestalt or griefwork or group therap$ or guided image$ or holistic therap$ or humanistic or hypnosis or hypnotherapy or hypnoti#zability or implosive therap$ or insight therap$ or integrative therap$ or integrative therap$ or interpersonal therap$ or Jungian or kleinian).mp.
12	(logotherap$ or logo therap$ or marathon group therap$ or marital therap$ or meditation or metacognitive or meta-cognitive or milieu or mind train$ or mindfulness or morita or multimodal therap$ or music therap$ or narrative therap$ or nondirective therap$ or non-directive therap$ or nondirective therap$ or non-specific therap$ or nonspecific therap$ or personal construct therap$ or person cent$ therap$ or pet therap$ or play therap$ or present cent$ therap$ or primal therap$ or problem focus$ therap$ or process experiential or psychoanaly$ or psychodrama or psychodynamic or psychotherap$).mp. [mp=title, abstract, original title, name of substance word, subject heading word, keyword heading word, protocol supplementary concept word, rare disease supplementary concept word, unique identifier, synonyms]
13	(rational emotive therap$ or reality therap$ or relationship therap$ or relaxation stress management or relaxation technique$ or relaxation therap$ or relaxation training or self analys$ or sensitivity training therap$ or sleep phase chronotherap$ or socioenvironment$ therap$ or social skill$ or

	sociotherap$ or solution focused therap$ or stress management or support group$ or (support adj3 psycho$) or supportive therap$ or systematic desensiti#ation or therapeutic techniqu$ or time limited therap$ or transference therap$ or validation therap$).mp.
14	Desensitization, Psychologic/
15	"Imagery (Psychotherapy)"/
16	randomi#ed controlled trial.pt.
17	controlled clinical trial.pt.
18	randomi#ed.ab.
19	trial.ti,ab.
20	(control adj group?).ab.
21	exp Pragmatic Clinical Trial/ or exp Clinical Trial/ or exp Randomized Controlled Trial/ or exp Controlled Clinical Trial/
22	exp Random Allocation/
23	(clin$ adj5 trial$).ti,ab.
24	((waitlist* or wait$ list* or treatment as usual or TAU) adj3 (control or group)).ab.
25	iCBT.mp.
26	iCBT.tw.
27	((developing or less developed or under developed or underdeveloped or middle income or low income or lower income) adj (countr* or nation* or world)).mp.
28	((transitional or developing or less developed or lesser developed or under developed or underdeveloped or middle income or low income or lower income) adj (economy or economies)).mp.
29	((low* adj (gdp or gnp or gross domestic or gross national)) or (lmic or lmics or lamics or lamic or third world or lami countries or lami country) or (transitional country or transitional countries)).mp.
30	exp Developing Countries/
31	(Afghanistan or Albania or Algeria or Angola or Argentina or Armenia or Armenian or Azerbaijan or Bangladesh or Benin or Byelarus or Byelorussian or Belarus or Belorussian or Belorussia or Belize or Bhutan or Bolivia or Bosnia or Herzegovina or Hercegovina or Botswana or Brazil or Bulgaria or Burkina Faso or Burkina Fasso or Upper Volta or Burundi or Urundi or Cambodia or Khmer Republic or Kampuchea or Cameroon or Cameroons or Cameron or Camerons or Cape Verde or Cabo Verde or Central African Republic or Chad or China or Colombia or Comoros or Comoro Islands or Comoros or Mayotte or Congo or Zaire or Costa Rica or Cote d Ivoire or Cote d'Ivoire or Ivory Coast or Croatia or Cuba or Djibouti or French

	Somaliland or Dominica or Dominican Republic or East Timor or East Timur or Timor Leste or Timor-Leste or Ecuador or Egypt or El Salvador or Equatorial Guinea or Eritrea or Ethiopia or Fiji or Gabon or Gabonese Republic or Gambia or Gaza or Georgia or Georgian Republic or Ghana or Gold Coast or Grenada or Guatemala or Guinea or Guinea-Bisseau or Guiana or Guyana or Haiti or Honduras or India or Indonesia or Iran or Iraq or Jamaica or Jordan or Kazakhstan or Kazakh or Kenya or Kiribati or Korea or Kosovo or Kyrgyzstan or Kyrgyz Republic or Kirghizia or Kyrgyz or Kirghiz or Kirgizstan or Lao or Lao PDR or Laos or Lebanon or Lesotho or Basutoland or Liberia or Libya or Macedonia or Madagascar or Malagasy or Malaysia or Malaya or Malay or Sabah or Sarawak or Malawi or Nyasaland or Mali or Marshall Islands or Mauritania or Mauritius or Agalega Islands or (Mexico not New Mexico) or Micronesia or Middle East or Maldives or Moldova or Moldovia or Moldovian or Mongolia or Montenegro or Morocco or Ifni or Mozambique or Myanmar or Myanma or Burma or Namibia or Nauru or Nepal or Netherlands Antilles or New Caledonia or Nicaragua or Niger or Nigeria or Mariana Islands or Pakistan or Palestine or Panama or Papua New Guinea or Paraguay or Peru or Philippines or Philipines or Phillipines or Phillippines or Romania or Rumania or Roumania or Russia or Russian or Rwanda or Ruanda or Saint Lucia or St Lucia or Saint Vincent or St Vincent or Grenadines or Samoa or Samoan Islands or Navigator Island or Navigator Islands or Sao Tome or Senegal or Serbia or Montenegro or Sierra Leone or South Africa or Sri Lanka or Ceylon or Solomon Islands or Somalia or Somaliland or South Africa or Sudan or South Sudan or Suriname or Surinam or Swaziland or Syria or Syrian Republic or Tajikistan or Tadzhikistan or Tadjikistan or Tadzhik or Tanzania or Thailand or Togo or Togolese or Tonga or Tunisia or Turkey or Turkmenistan or Turkmen or Tuvalu or Venezuela or Uganda or Ukraine or USSR or Soviet Union or Union of Soviet Socialist Republics or Uzbekistan or Uzbek or Vanuatu or New Hebrides or Venezuela or Vietnam or Viet Nam or West Bank or Yemen or Yugoslavia or Zambia or Zimbabwe or Rhodesia).mp.
32	27 or 28 or 29 or 30 or 31
33	exp Mental Disorders/ or exp Mental illness/
34	exp Mental Health/
35	exp Mentally Ill Persons/ or exp Community Mental Health Services/
36	exp Depression/ or exp Depression, Postpartum/
37	exp Neurotic Disorders/
38	exp Depressive Disorder/ or exp Stress Disorders, Traumatic/ or exp Stress Disorders, Post-Traumatic/ or exp Stress Disorders, Traumatic, Acute/ or exp Stress, Psychological/
39	exp Obsessive-Compulsive Disorder/ or exp Obsessive Behavior/ or exp Anxiety/ or exp Anxiety Disorders/ or exp somatoform disorder/
40	((common adj mental adj disorders) or anxiety or depression or distress or (panic adj disorder) or (depressive adj disorder) or (depressive adj symptoms) or (anxious adj symptoms) or (somatization adj symptoms) or (somatisation adj symptoms) or (somatization adj disorder) or (somatisation adj disorder) or (somatoform adj symptoms)).mp. [mp=title, abstract, original title, name of substance word, subject heading word, keyword heading word, protocol supplementary concept word, rare disease supplementary concept word, unique identifier, synonyms]

41	(obsessive or compulsive).mp. [mp=title, abstract, original title, name of substance word, subject heading word, keyword heading word, protocol supplementary concept word, rare disease supplementary concept word, unique identifier, synonyms]
42	(Neurosis or psychoneurosis).mp. [mp=title, abstract, original title, name of substance word, subject heading word, keyword heading word, protocol supplementary concept word, rare disease supplementary concept word, unique identifier, synonyms]
43	((mood or neurotic or stress or reactive or somatoform or somatization or somatisation or anxiety or phobic or obsessive-compulsive or adjustment or dissociat$) adj2 disorder$).mp. [mp=title, abstract, original title, name of substance word, subject heading word, keyword heading word, protocol supplementary concept word, rare disease supplementary concept word, unique identifier, synonyms]
44	16 or 17 or 18 or 19 or 20 or 21 or 22 or 23 or 24
45	33 or 34 or 35 or 36 or 37 or 38 or 39 or 40 or 41 or 42 or 43
46	1 or 2 or 3 or 4 or 5 or 6 or 7 or 8 or 9 or 10 or 11 or 12 or 13 or 14 or 15 or 25 or 26
47	32 and 44 and 45 and 46

2: Timing and safety characteristics of included records

CMHW=community mental health worker. EUC=enhanced usual care. IPV=intimate partner violence. mhGAP=WHO Mental Health Gap Action Programme. MINI=Mini-International Neuropsychiatric Interview. NET=narrative exposure therapy. NGO=non-governmental organisation. PTSD=Post-Traumatic Stress Disorder. SSQ-14=Shona Symptom Questionnaire.

	**Timing**	**Safety aspects**
Bass et al (2016)	June, 2009, to June, 2010 (screening)	CMHWs adhered to ethical standards (do no harm, maintain professional relations, and boundaries); they were advised to monitor their own feelings, follow self-care strategies, take up professional consultation and support, and maintain work-life balance; control participants were advised to contact CMHWs at any time if their symptoms worsened; such participants were assessed for consideration of referral and transfer to a psychiatrist or trauma rehabilitation centre
Bolton et el (2014)	June, 2009, to June, 2010 (recruitment)	Control participants were advised to contact CMHWs at any time if their symptoms worsened substantially; such participants were assessed for consideration of referral and transfer to a psychiatrist or torture treatment centre
Brown et al (2018), Tol et al (2018), and Tol et al (2019)	March to April, 2017 (recruitment)	Group peer review after each session covering difficulties in delivery, concerns, adverse events; serious adverse events reported to a data safety and monitoring board to act if needed
Bryant et al (2017)	April 15, to Aug 20, 2015 (screening), Jan 16, 2016 (final follow-up)	Cases of threat of harm or self-harm referred to local services; psychiatric crises (imminent suicidal risk as defined by suicidal plan) or need for acute protection referred to local advisory board and participant referred to appropriate services, including local hospitals providing psychiatric care; adverse reactions monitored and recorded throughout
Chibanda et al (2016)	Recruitment to final follow-up: Sept 1, 2014, to May 25, 2015	Individuals excluded for psychiatric reasons were referred to a tertiary health-care facility; a trained, supervised lay health worker attached to the clinic referred participants

		who were not improving or reported suicidal ideation to a mental health trained supervisor for reassessment and management; intervention group participants were assessed by a psychiatrist if worsened SSQ-14 score after three sessions
Ertl et al (2011)	Recruitment to final follow-up: November, 2007, to October, 2009	Waiting list participants who scored very high levels of suicidal ideation received suicide intervention; waiting list and academic catch-up participants still exhibiting PTSD at 12 months' follow-up were offered NET
Fuhr et al (2019)	Oct 24, 2014, to June 24, 2016 (eligibility assessment), May 27, 2017 (final treatment)	Sakhi training included dealing with difficult situations, recognising worsening symptoms and serious adverse events; Sakhis had a maximum case load of four women at any one time
Grundlingh et al (2017)	June 15–16, 2014 (recruitment),<br>June 17, to July 11, 2014 (baseline to endline assessment)	All participants received contact details for external support services and were referred to additional counselling if severe secondary trauma or vicarious trauma detected; care taken not to pressure individuals to disclose personal experiences; option to write experiences on paper
Lund et al (2019)	October, 2013 to October, 2014 (enrolment);<br>May 2016 (final follow-up)	Participants scoring >16 on MINI referred to community health centre psychiatric nurses and local district hospital if further management indicated; control participants provided with contact details of relevant health services and NGOs if signs of abuse or other social difficulties; data safety and monitoring board monitored serious adverse events for which there was a response protocol; initial training covered staff burnout; staff counselling available if needed
Patel et al (2017) and Weobong et al (2017)	Oct 28, 2013, to July 29, 2015 (enrolment and randomisation)	Participants who did not respond could be referred for specialist care; EUC included information about referral; study overseen by a data and safety monitoring committee; serious adverse event data collected.
Sikander et al (2018)	Oct 15, 2014, to Feb 25, 2016 (village randomisation)	Overseen by data safety and monitoring board; data collected on serious adverse events; mhGAP EUC contained guidelines about referral of women at risk of suicide

Steinert et al (2017)	May, 2012, to June, 2014 (recruitment)	Not mentioned
Latif et al (2017)	30–35 weeks; dates not provided	Not mentioned
Orang et al (2018)	Not mentioned	Interviews and treatment sessions at quiet, private locations, close to home; women provided with information and contact details about police, legal, medical and social work services and encouraged to seek formal and informal family, social and legal support; 1–2 NET sessions focused on safety issues and current violence, including coping skill enhancement, safety planning, human rights education; each NET session began with brief discussion of IPV occurrences or marital arguments during the previous week; where recent severe IPV had occurred, sessions were cancelled, postponed, or refocused on safety planning and acknowledgement of suffering; three participants dropped out owing to extremely high IPV occurrences; three discontinued NET owing to distress arising from IPV discussion

3: Independent samples t-tests for the difference between baseline mean CMD scores for included studies in meta-analyses of anxiety (A), PTSD (B), depression (C) and psychological distress (D) symptoms

A

First author, Year	Measure	Participants reporting no IPV			Participants reporting IPV			t	p
		N	Mean	Standard deviation	N	Mean	Standard deviation		
Chibanda 2016	GAD7	69	9·9	5·3	161	11·3	5	-1·91	0·057
Bass 2016	HSCL-25anx	27	12·85	5·22	27	15·44	4·37	-1·98	0·053
Bolton 2014a	HSCL-25anx	43	14·44	5·97	22	16	4·32	-1·09	0·28
Bolton 2014b	HSCL-25anx	40	13·78	5·54	18	16·5	4·19	-1·86	0·068
Steinert 2017	HSCL-25anx	25	2·96	0·7	9	3·02	0·61	-0·23	0·82

B

First author, Year	Measure	Participants reporting no IPV			Participants reporting IPV			t	p
		N	Mean	Standard deviation	N	Mean	Standard deviation		
Tol 2019	PCL-6	219	21·82	4·82	99	22·39	4·32	-1·01	0·31
Ertl 2011a	CAPS	1	84	0	15	68	15	N/A	N/A
Ertl 2011b	CAPS	8	66	11·87	11	60·18	15·38	0·89	0·39
Bryant 2017	PCL-5	59	26·73	20·42	150	36·23	19·11	-3·17	0·0017*
Bass 2016	HTQptsd	27	34·30	12·73	27	43·44	9·79	-2·96	0·0046*
Bolton 2014a	HTQptsd	43	40·23	14·24	22	43·59	13·80	-0·91	0·37
Bolton 2014b	HTQptsd	40	42·58	13·46	18	46·33	10·57	-1·05	0·30
Steinert 2017	HTQptsd	25	3·37	0·45	9	3·4	0·38	-0·18	0·86

C

First author, Year	Measure	Participants reporting no IPV			Participants reporting IPV			t	p
		N	Mean	Standard deviation	N	Mean	Standard deviation		
Lund 2019	HDRS	174	15·19	4·54	31	18	5·93	-3·02	0·0028*
Ertl 2011a	MINI	1	6	0	15	3	3·14	N/A	N/A
Ertl 2011b	MINI	8	3·38	2·45	11	2·73	3·07	0·49	0·63
Tol 2019	PHQ9	219	14·93	4·75	99	15·6	4·65	-1·17	0·24
Chibanda 2016	PHQ9	69	11·1	5·5	161	13·8	5·5	-3·41	0·0008*
Patel 2016	PHQ9	75	17·6	2·69	28	18·43	3·08	-1·34	0·18

Fuhr 2018	PHQ9	124	13·44	3·42	16	15·38	3·79	-2·11	0·037*
Sikander 2018	PHQ9	245	14·73	3·64	30	16·6	4·17	-2·61	0·0095*
Steinert 2017	HSCL-25dep	25	3·04	0·66	9	3·34	0·39	-1·28	0·21
Bass 2016	HSCL-25dep	27	31·04	6·95	27	33·37	8·19	-1·13	0·26
Bolton 2014a	HSCL-25dep	43	33·61	8·19	22	39·09	10·20	-2·35	0·022*
Bolton 2014b	HSCL-25dep	40	35·23	7·75	18	36·33	7·11	-0·52	0·61

D

**First author, Year**	**Measure**	**Participants reporting no IPV**			**Participants reporting IPV**			**t**	**p**
		**N**	**Mean**	**Standard deviation**	**N**	**Mean**	**Standard deviation**		
Chibanda 2016	SSQ-14	69	10·1	1·1	161	10·8	1·4	-3·69	0·0003*
Tol 2019	Kessler 6	232	16·44	4·09	99	16·73	4·21	-0·59	0·56
Grundlingh 2017	SRQ-20	12	2·5	1·09	3	2·33	2·31	0·19	0·85
Bryant 2017	GHQ-12	59	18·24	6·29	150	10·67	5·82	-1·56	0·12

4: Cochrane Risk of Bias Tool assessments

	Random sequence generation (selection bias)	Allocation concealment (selection bias)	Participant/ personnel blinding (performance bias)	Primary outcome assessment blinding (detection bias)	Secondary outcome assessment blinding (detection bias)	Incomplete primary outcome data (attrition bias)	Incomplete secondary outcome data (attrition bias)	Selective reporting (reporting bias)	Other bias	Overall risk of bias
**Bass et al. 2016**	Low	Low	High	High	High	Low	Low	Unclear	High	Moderate
**Bolton et al. 2014**	Low	Unclear	High	High	High	Low	Low	Unclear	High	Moderate
**Tol et al. 2019**	Low	Unclear	High	Low	Low	Low	Low	Low	High	Moderate
**Bryant et al. 2017**	Low	Low	High	Low	Low	Low	Low	Low	High	Moderate
**Chibanda et al. 2016**	Low	Low	High	Low	Low	Low	Low	Low	Low	Low
**Ertl et al. 2011**	Unclear	Unclear	High	Low	Low	Low	Low	Low	Low	Moderate
**Fuhr et al. 2019**	Low	Low	High	Low	Low	Low	Low	High	Low	Moderate
**Grundlingh et al. 2017**	Low	Low	High	Low	Low	Low	Low	Low	Low	Low
**Lund et al. 2019**	Low	Low	High	Low	Low	Unclear	Unclear	Unclear	Unclear	Moderate
**Patel et al. 2017**	Low	Low	High	Low	Low	Low	Low	Low	Low	Low
**Sikander et al. 2018**	Low	Low	High	Low	Low	Low	Low	Low	Low	Low
**Steinert et al. 2017**	High	Low	High	Low	Low	High	High	Unclear	Low	Moderate

Key	
(red)	High risk of bias
(amber)	Moderate risk of bias
(green)	Low risk of bias
(grey)	Unclear

5: Funnel Plot for studies included in the meta-analysis of depression symptoms

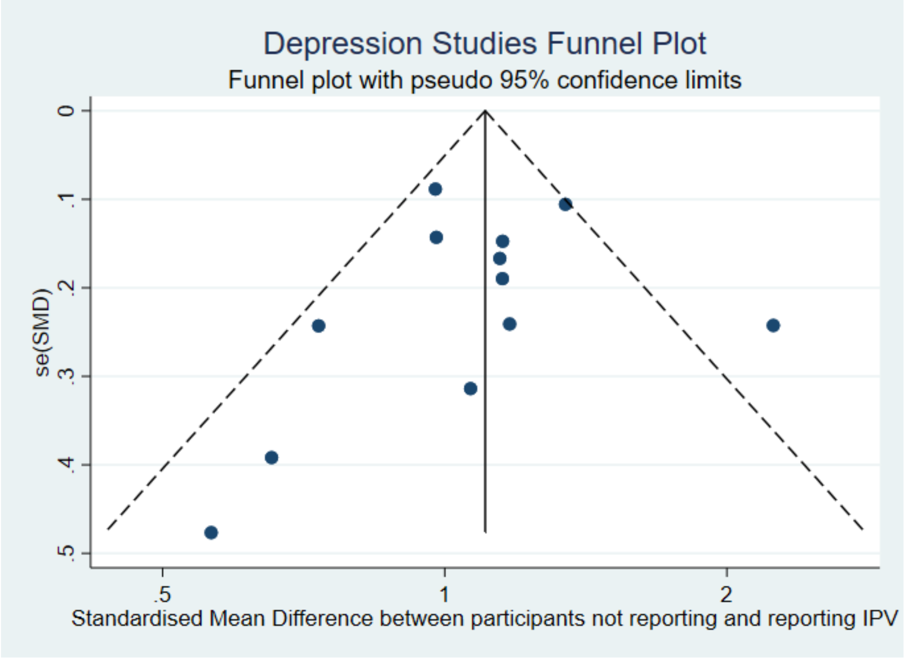

6: Random-effects meta-analyses of the difference in psychological intervention study effect sizes (dSMD) between women who did and women who did not report exposure to intimate partner violence (IPV), for anxiety (A), depression (B), PTSD (C) and psychological distress (D) symptoms, comparing generic and explicitly trauma focused interventions

A

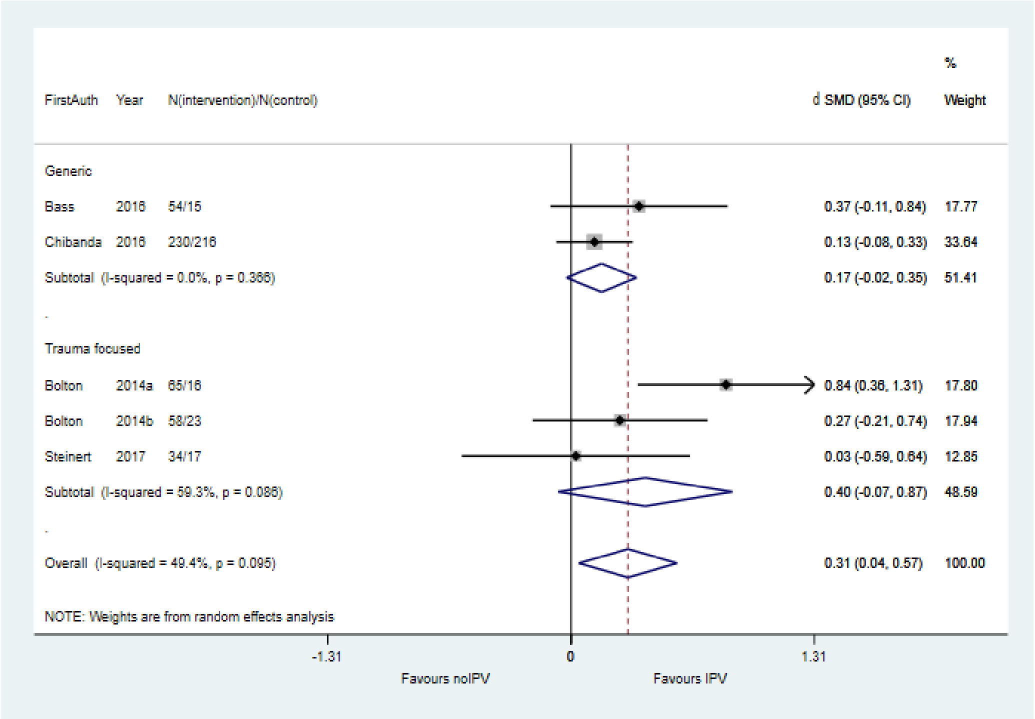

B

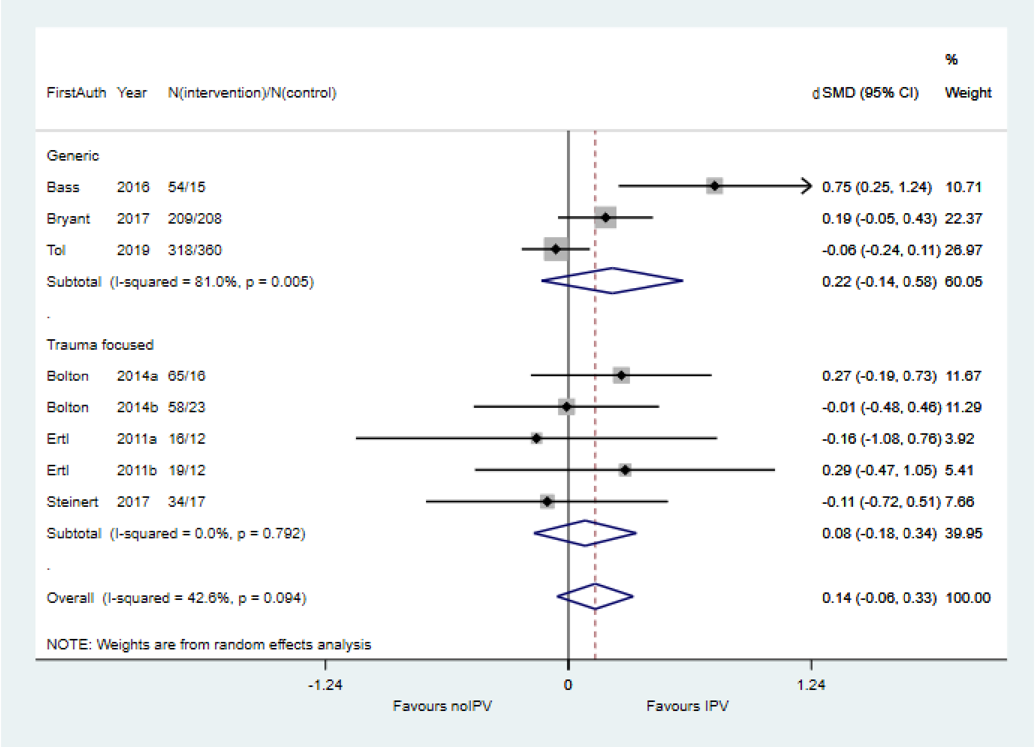

C

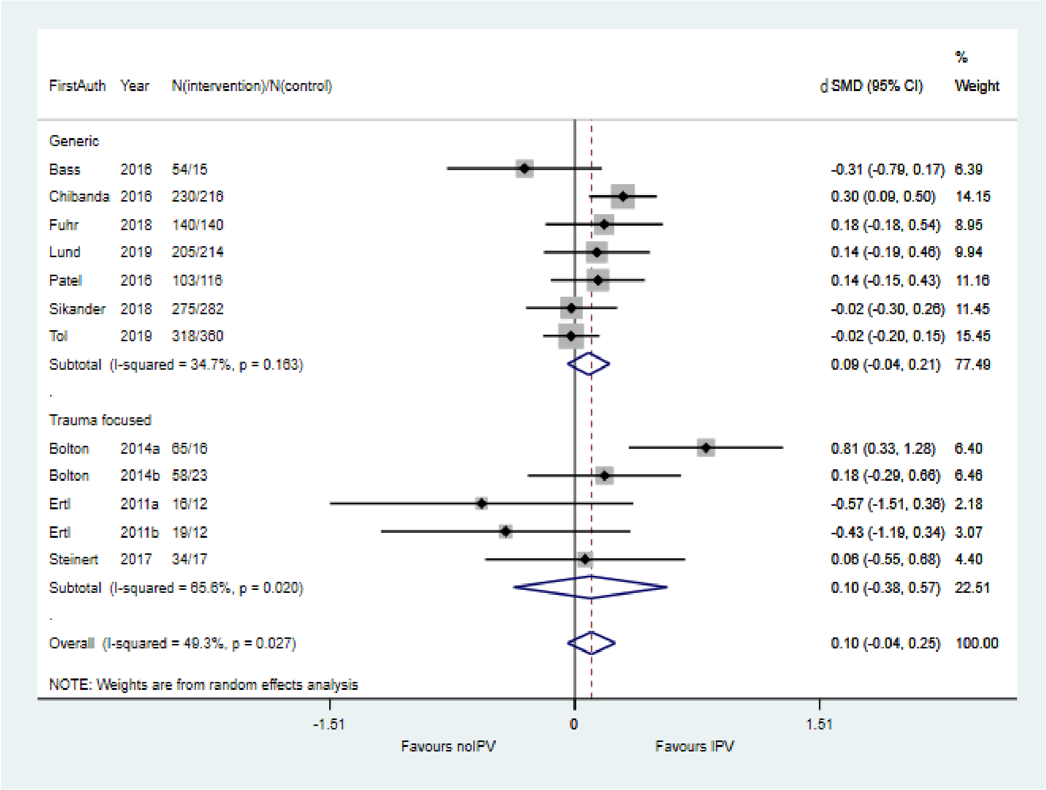
FirstAuth Year N(intervention)/N(control)
d SMD (95% CI)
% Weight
Generic
Bass 2016 54/15 -0.31 (-0.79, 0.17) 6.39
Chibanda 2016 230/216 0.30 (0.09, 0.50) 14.15
Fuhr 2018 140/140 0.18 (-0.18, 0.54) 8.95
Lund 2019 205/214 0.14 (-0.19, 0.46) 9.94
Patel 2016 103/116 0.14 (-0.15, 0.43) 11.16
Sikander 2018 275/282 -0.02 (-0.30, 0.26) 11.45
Tol 2019 318/360 -0.02 (-0.20, 0.15) 15.45
Subtotal (I-squared = 34.7%, p = 0.163) 0.09 (-0.04, 0.21) 77.49
Trauma focused
Bolton 2014a 65/16 0.81 (0.33, 1.28) 6.40
Bolton 2014b 58/23 0.18 (-0.29, 0.66) 6.46
Ertl 2011a 16/12 -0.57 (-1.51, 0.36) 2.18
Ertl 2011b 19/12 -0.43 (-1.19, 0.34) 3.07
Steinert 2017 34/17 0.06 (-0.55, 0.68) 4.40
Subtotal (I-squared = 65.6%, p = 0.020) 0.10 (-0.38, 0.57) 22.51
Overall (I-squared = 49.3%, p = 0.027) 0.10 (-0.04, 0.25) 100.00
NOTE: Weights are from random effects analysis
-1.51
0
1.51
Favours noIPV
Favours IPV

D

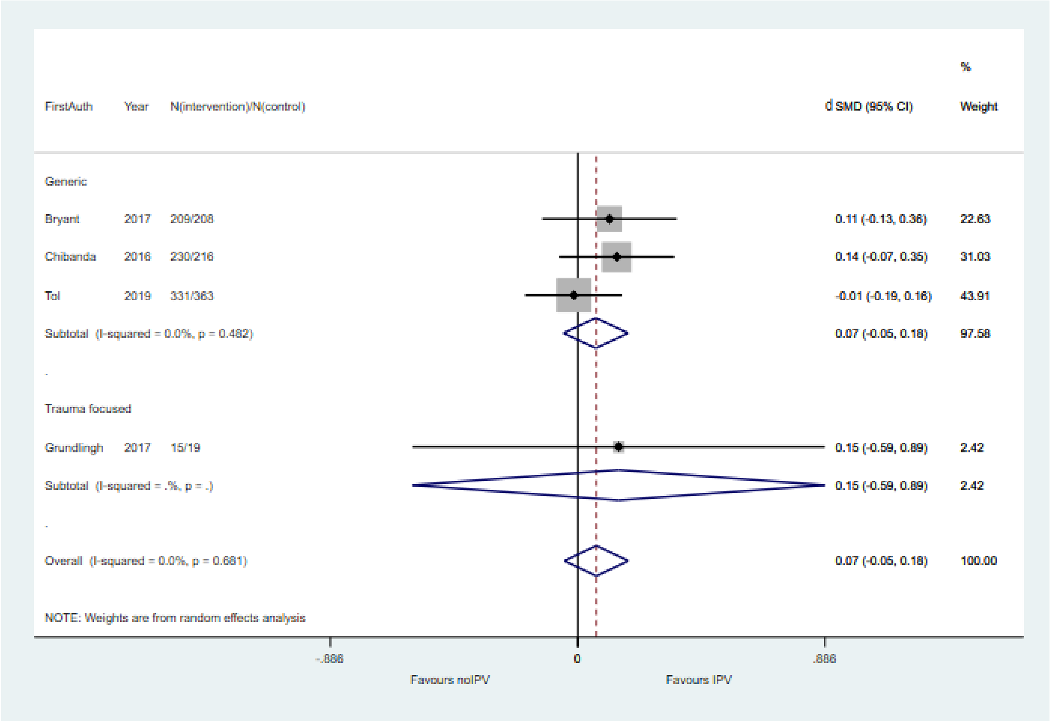
FirstAuth Year N(intervention)/N(control)
d SMD (95% CI)
% Weight
Generic
Bryant 2017 209/208 0.11 (-0.13, 0.36) 22.63
Chibanda 2016 230/216 0.14 (-0.07, 0.35) 31.03
Tol 2019 331/363 -0.01 (-0.19, 0.16) 43.91
Subtotal (I-squared = 0.0%, p = 0.482) 0.07 (-0.05, 0.18) 97.58
Trauma focused
Grundlingh 2017 15/19 0.15 (-0.59, 0.89) 2.42
Subtotal (I-squared = .%, p = .) 0.15 (-0.59, 0.89) 2.42
Overall (I-squared = 0.0%, p = 0.681) 0.07 (-0.05, 0.18) 100.00
NOTE: Weights are from random effects analysis
-.886
0
.886
Favours noIPV
Favours IPV

7: Sensitivity analyses reviewing changes to pooled dSMD estimates when one study was removed from each meta-analysis at a time, for anxiety (A), PTSD (B), depression (C) and psychological distress (D) symptoms

A

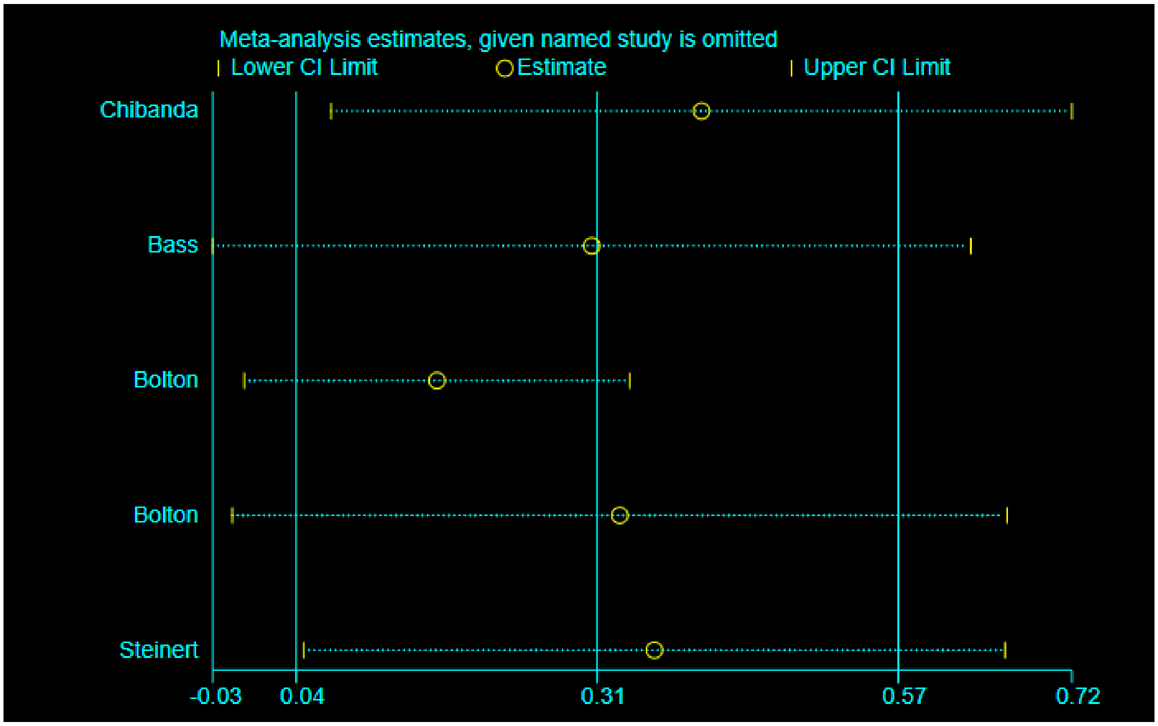

B

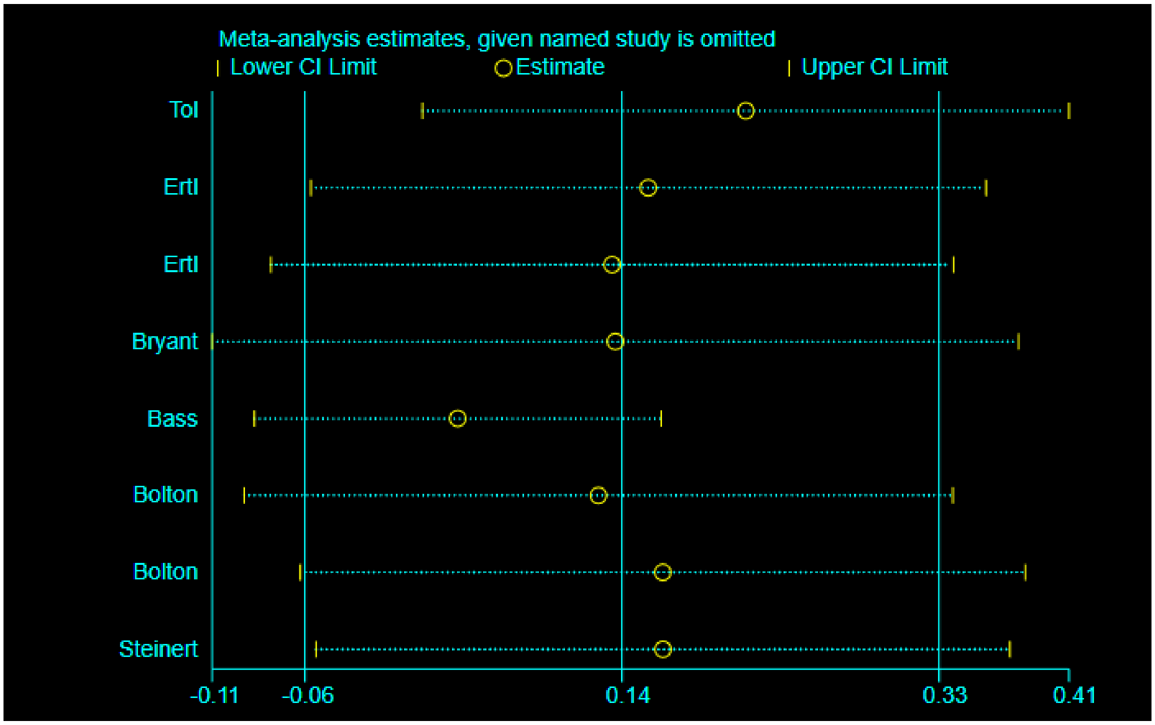

C

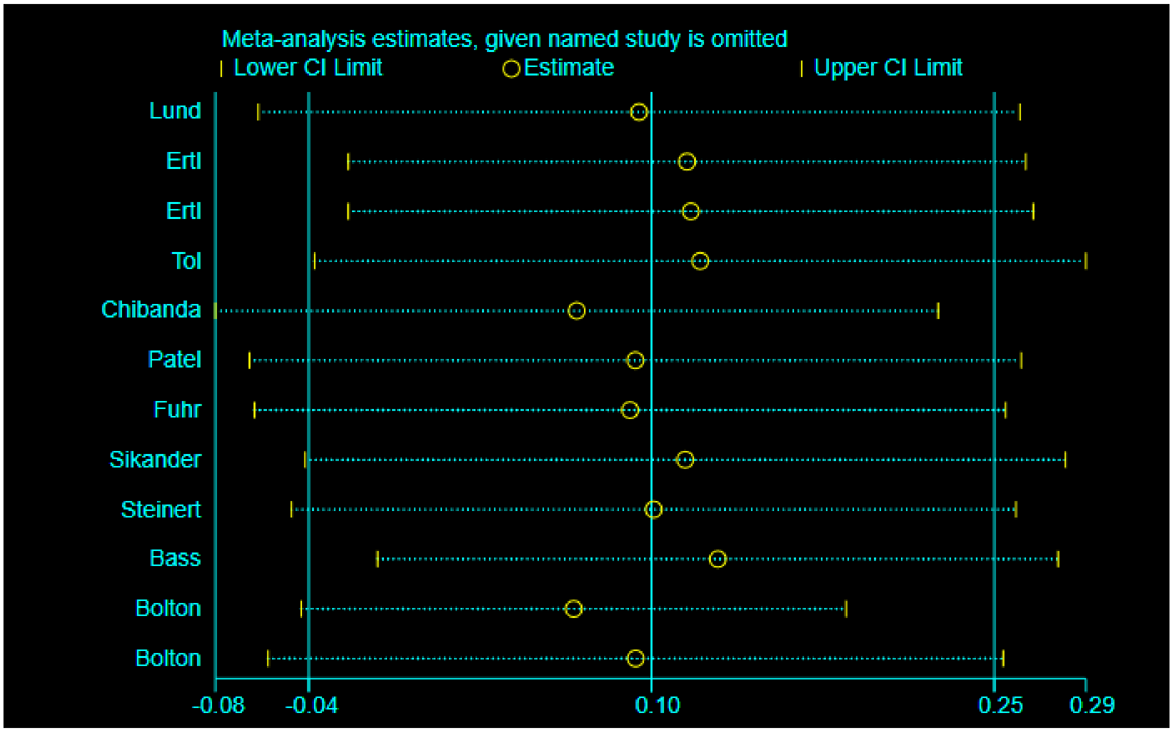
Meta-analysis estimates, given named study is omitted
Lower CI Limit
Estimate
Upper CI Limit
Lund
Ertl
Ertl
Tol
Chibanda
Patel
Fuhr
Sikander
Steinert
Bass
Bolton
Bolton
-0.08
-0.04
0.10
0.25
0.29

D

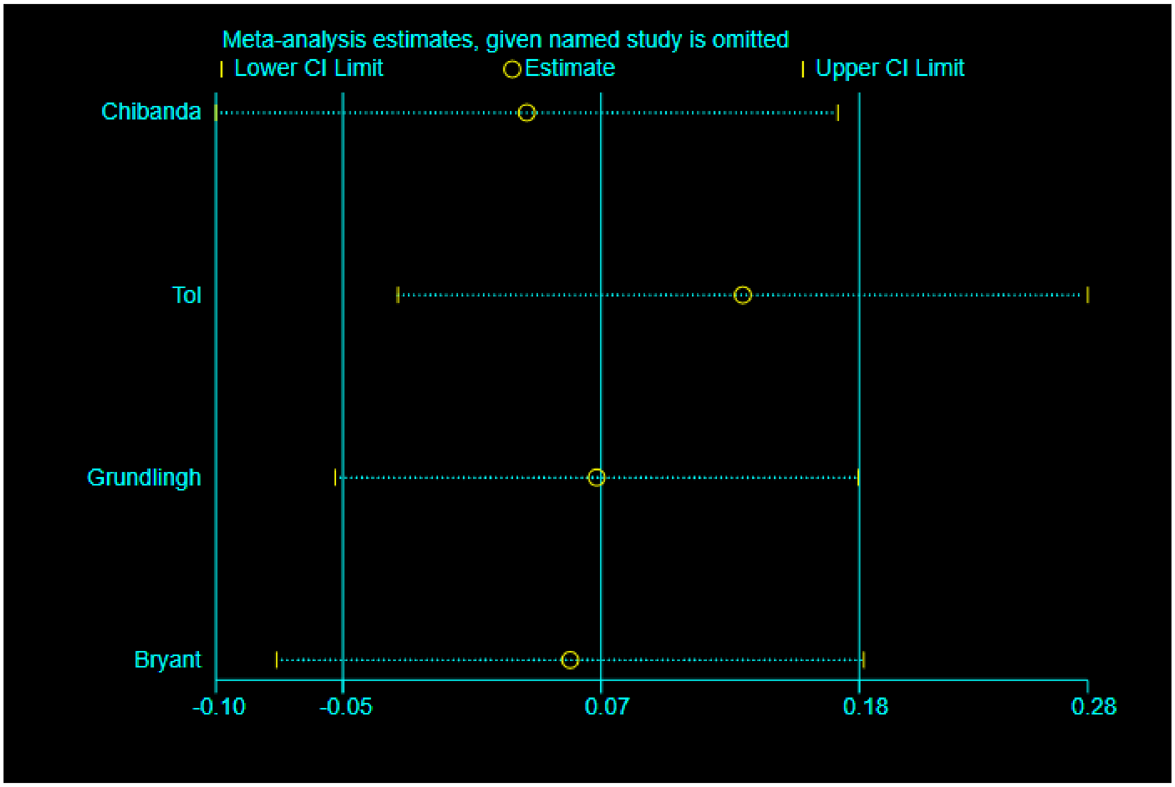
Meta-analysis estimates, given named study is omitted
Lower CI Limit
Estimate
Upper CI Limit
Chibanda
Tol
Grundlingh
Bryant
-0.10
-0.05
0.07
0.18
0.28

8: Comparison between country(1) or regional IPV prevalence(2) and study prevalence or incidence

Country	Relevant studies	National lifetime physical and/or sexual IPV prevalence (%)	National physical and/or sexual IPV incidence in the last 12 months (%)	Regional lifetime physical and/or sexual IPV among ever-partnered women (%)	Study IPV prevalence or incidence (%)
Cambodia	Steinert et al. 2017	21	8	37·7 (South-East Asia)	27·5 (prevalence)
India	Fuhr et al. 2019	29	22		13·2 (3 month incidence)
	Patel et al. 2017				31·5 (prevalence)
Pakistan	Sikander et al. 2018	Not available	Not available		12·8 (3 month incidence)
Iraq	Bass et al. 2016	Not available	Not available	37 (Eastern Mediterranean)	46·4 (prevalence)
	Bolton et al. 2014a				34·6 (prevalence)
	Bolton et al. 2014b				30·9 (prevalence)
Kenya	Bryant et al. 2017	39	26	36·6 (Africa)	71·0 (prevalence)
South Africa	Lund et al. 2019	Not available	Not available		13·8 (3 month incidence)
Uganda	Tol et al. 2019	50	30		29·9 (12 month incidence)
	Ertl et al. 2011a				78·6 (prevalence)
	Ertl et al. 2011b				58·1 (prevalence)
	Grundlingh et al. 2017				29·4 (12 month incidence)
Zimbabwe	Chibanda et al. 2016	35	20		71·7 (6 month incidence)

REFERENCES

1. UN_Women. Global Database on Violence against Women 2016 [Available from: http://evaw-global-database.unwomen.org/en/countries.

2. WHO. Global and regional estimates of violence against women: prevalence and health effects of intimate partner violence and non-partner sexual violence: World Health Organization; 2013.

9: PRISMA 2009 Checklist

Section/topic	#	Checklist item	Reported on page
**TITLE**			
Title	1	Identify the report as a systematic review, meta-analysis, or both.	1
**ABSTRACT**			
Structured summary	2	Provide a structured summary including, as applicable: background; objectives; data sources; study eligibility criteria, participants, and interventions; study appraisal and synthesis methods; results; limitations; conclusions and implications of key findings; systematic review registration number.	2
**INTRODUCTION**			
Rationale	3	Describe the rationale for the review in the context of what is already known.	4
Objectives	4	Provide an explicit statement of questions being addressed with reference to participants, interventions, comparisons, outcomes, and study design (PICOS).	5
**METHODS**			
Protocol and registration	5	Indicate if a review protocol exists, if and where it can be accessed (e.g., Web address), and, if available, provide registration information including registration number.	5
Eligibility criteria	6	Specify study characteristics (e.g., PICOS, length of follow-up) and report characteristics (e.g., years considered, language, publication status) used as criteria for eligibility, giving rationale.	5
Information sources	7	Describe all information sources (e.g., databases with dates of coverage, contact with study authors to identify additional studies) in the search and date last searched.	5
Search	8	Present full electronic search strategy for at least one database, including any limits used, such that it could be repeated.	Appendix 1
Study selection	9	State the process for selecting studies (i.e., screening, eligibility, included in systematic review, and, if applicable, included in the meta-analysis).	5
Data collection process	10	Describe method of data extraction from reports (e.g., piloted forms, independently, in duplicate) and any processes for obtaining and confirming data from investigators.	5
Data items	11	List and define all variables for which data were sought (e.g., PICOS, funding sources) and any assumptions and simplifications made.	5
Risk of bias in individual studies	12	Describe methods used for assessing risk of bias of individual studies (including specification of whether this was done at the study or outcome level), and how this information is to be used in any data synthesis.	6
Summary measures	13	State the principal summary measures (e.g., risk ratio, difference in means).	5
Synthesis of results	14	Describe the methods of handling data and combining results of studies, if done, including measures of consistency (e.g., I^2) for each meta-analysis.	6

Section/topic	#	Checklist item	Reported on page
Risk of bias across studies	15	Specify any assessment of risk of bias that may affect the cumulative evidence (e.g., publication bias, selective reporting within studies).	6
Additional analyses	16	Describe methods of additional analyses (e.g., sensitivity or subgroup analyses, meta-regression), if done, indicating which were pre-specified.	6
**RESULTS**			
Study selection	17	Give numbers of studies screened, assessed for eligibility, and included in the review, with reasons for exclusions at each stage, ideally with a flow diagram.	6-9
Study characteristics	18	For each study, present characteristics for which data were extracted (e.g., study size, PICOS, follow-up period) and provide the citations.	Table 1
Risk of bias within studies	19	Present data on risk of bias of each study and, if available, any outcome level assessment (see item 12).	Appendix 3
Results of individual studies	20	For all outcomes considered (benefits or harms), present, for each study: (a) simple summary data for each intervention group (b) effect estimates and confidence intervals, ideally with a forest plot.	7-15
Synthesis of results	21	Present results of each meta-analysis done, including confidence intervals and measures of consistency.	7-8
Risk of bias across studies	22	Present results of any assessment of risk of bias across studies (see Item 15).	7
Additional analysis	23	Give results of additional analyses, if done (e.g., sensitivity or subgroup analyses, meta-regression [see Item 16]).	8
**DISCUSSION**			
Summary of evidence	24	Summarize the main findings including the strength of evidence for each main outcome; consider their relevance to key groups (e.g., healthcare providers, users, and policy makers).	16-19
Limitations	25	Discuss limitations at study and outcome level (e.g., risk of bias), and at review-level (e.g., incomplete retrieval of identified research, reporting bias).	17-18
Conclusions	26	Provide a general interpretation of the results in the context of other evidence, and implications for future research.	19
**FUNDING**			
Funding	27	Describe sources of funding for the systematic review and other support (e.g., supply of data); role of funders for the systematic review.	20

From: Moher D, Liberati A, Tetzlaff J, Altman DG, The PRISMA Group (2009). Preferred Reporting Items for Systematic Reviews and Meta-Analyses: The PRISMA Statement. PLoS Med 6(7): e1000097. doi:10.1371/journal.pmed1000097 For more information, visit: **www.prisma-statement.org**.
